# Transitions in wheat endosperm metabolism upon transcriptional induction of oil accumulation by oat endosperm WRINKLED1

**DOI:** 10.1186/s12870-020-02438-9

**Published:** 2020-05-25

**Authors:** Åsa Grimberg, Mark Wilkinson, Per Snell, Rebecca P. De Vos, Irene González-Thuillier, Ahmed Tawfike, Jane L. Ward, Anders S. Carlsson, Peter Shewry, Per Hofvander

**Affiliations:** 1 Department of Plant Breeding, Swedish University of Agricultural Sciences, SE-23053 Alnarp, Sweden; 2 Department of Plant Sciences, Rothamsted Research, Harpenden, AL5 2JQ UK; 3 Current address: MariboHilleshög Research AB, Box 302, 261 23 Landskrona, Sweden; 4 Department of Computational and Analytical Sciences, Rothamsted Research, Harpenden, AL5 2JQ UK

**Keywords:** Aleurone, Carbon allocation, Cereal endosperm, Oil biosynthesis, Transcriptional regulation, Triacylglycerol, Wheat, WRINKLED1

## Abstract

**Background:**

Cereal grains, including wheat (*Triticum aestivum* L.), are major sources of food and feed, with wheat being dominant in temperate zones. These end uses exploit the storage reserves in the starchy endosperm of the grain, with starch being the major storage component in most cereal species. However, oats (*Avena sativa* L.) differs in that the starchy endosperm stores significant amounts of oil. Understanding the control of carbon allocation between groups of storage compounds, such as starch and oil, is therefore important for understanding the composition and hence end use quality of cereals. WRINKLED1 is a transcription factor known to induce triacylglycerol (TAG; oil) accumulation in several plant storage tissues.

**Results:**

An oat endosperm homolog of *WRI1* (*AsWRI1*) expressed from the endosperm-specific *HMW1Dx5* promoter resulted in drastic changes in carbon allocation in wheat grains, with reduced seed weight and a wrinkled seed phenotype. The starch content of mature grain endosperms of *AsWRI1*-wheat was reduced compared to controls (from 62 to 22% by dry weight (dw)), TAG was increased by up to nine-fold (from 0.7 to 6.4% oil by dw) and sucrose from 1.5 to 10% by dw. Expression of *AsWRI1* in wheat grains also resulted in multiple layers of elongated peripheral aleurone cells. RNA-sequencing, lipid analyses, and pulse-chase experiments using ^14^C-sucrose indicated that futile cycling of fatty acids could be a limitation for oil accumulation.

**Conclusions:**

Our data show that expression of oat endosperm *WRI1* in the wheat endosperm results in changes in metabolism which could underpin the application of biotechnology to manipulate grain composition. In particular, the striking effect on starch synthesis in the wheat endosperm indicates that an important indirect role of WRI1 is to divert carbon allocation away from starch biosynthesis in plant storage tissues that accumulate oil.

## Background

Cereal grains are the world’s most important plant sources of food and feed, with wheat providing between 20 and 50% of the total calories consumed by humans in wheat-growing areas [1]. The major part of the grain is the endosperm, which accounts for about 90% of the wheat (*Triticum aestivum*) grain dry weight (dw) and comprises a single layer of outer aleurone cells (about 6.5% of grain dw) surrounding the starchy endosperm (about 83% of grain dw) which is the major grain storage tissue [2]. Small amounts of oil (triacylglycerols; TAG) are stored in the embryo and aleurone cells that, together with minor amounts in the starchy endosperm, contribute to a total lipid content of approximately 3% of grain dw [3].

By contrast, oats (*Avena sativa*) is unique among cereals in storing significant amounts of oil (up to 18% by grain dw) most of which is deposited in the starchy endosperm [4–7]. Increasing the oil content of cereal grains is of interest due to the increased global demands for vegetable oil production and also to improve the nutritional quality which could broaden their use [8, 9]. The development of high-oil maize (*Zea mays* L.) varieties was mainly due to increased proportions of embryo and embryo oil content [10]. However, the wheat embryo is not widely consumed, being lost in the bran fraction when the grain is milled to produce white flour (which is still the major form in which wheat is consumed in most countries such as the UK where it accounts for almost 90% of breadmaking flour [11]). It is therefore of interest to explore strategies to modify carbon allocation to increase the oil content of the starchy endosperm.

Transcription factors that can modulate whole genetic programs in metabolic switches are attractive tools for reprogramming of plant carbon metabolism. WRINKLED1 (WRI1) is a transcription factor known to have key importance for seed oil accumulation. It was first identified in Arabidopsis [12, 13] and homologs have since been identified in storage tissues of other plants [14–19], including the oat starchy endosperm [20–22]. The TAG biosynthesis pathway in plants involves multiple subcellular organelles, with fatty acids being produced de novo in the plastid and transported into the endoplasmic reticulum where they are esterified to a glycerol backbone to form TAG [23, 24]. The gene targets of WRI1 are known to be involved in glycolysis and fatty acid synthesis and give increased availability of carbon precursors for TAG synthesis [13, 25, 26]. Overexpression of *WRI1* in seeds of several dicot species that already accumulate high levels of oil has resulted in increases in oil content of 10–40% [12, 14, 19, 27, 28]. It has also been shown that oil accumulation in potato (*Solanum tuberosum* L.) tuber parenchyma is induced by tuber-specific expression of Arabidopsis *WRI1* [29]. By contrast, attempts to increase the oil content of maize by extending the expression of a maize embryo *WRI1* homolog to the starchy endosperm from the maize endosperm ZEIN promoter did not result in oil accumulation in the tissue [30].

Here, we determine the molecular, biochemical, and morphological changes in developing wheat grains in which oil accumulation is induced by overexpression of an oat endosperm homolog to *WRI1* from a starchy endosperm-specific promoter. This study not only demonstrates the feasibility of producing novel types of wheat with high oil content in white flour, but also contributes to our understanding of the regulation of carbon allocation in the cereal endosperm.

## Results

### Wrinkled seed phenotype, reduced seed weight, and multiple layers of aleurone cells

**Fig. 1:**
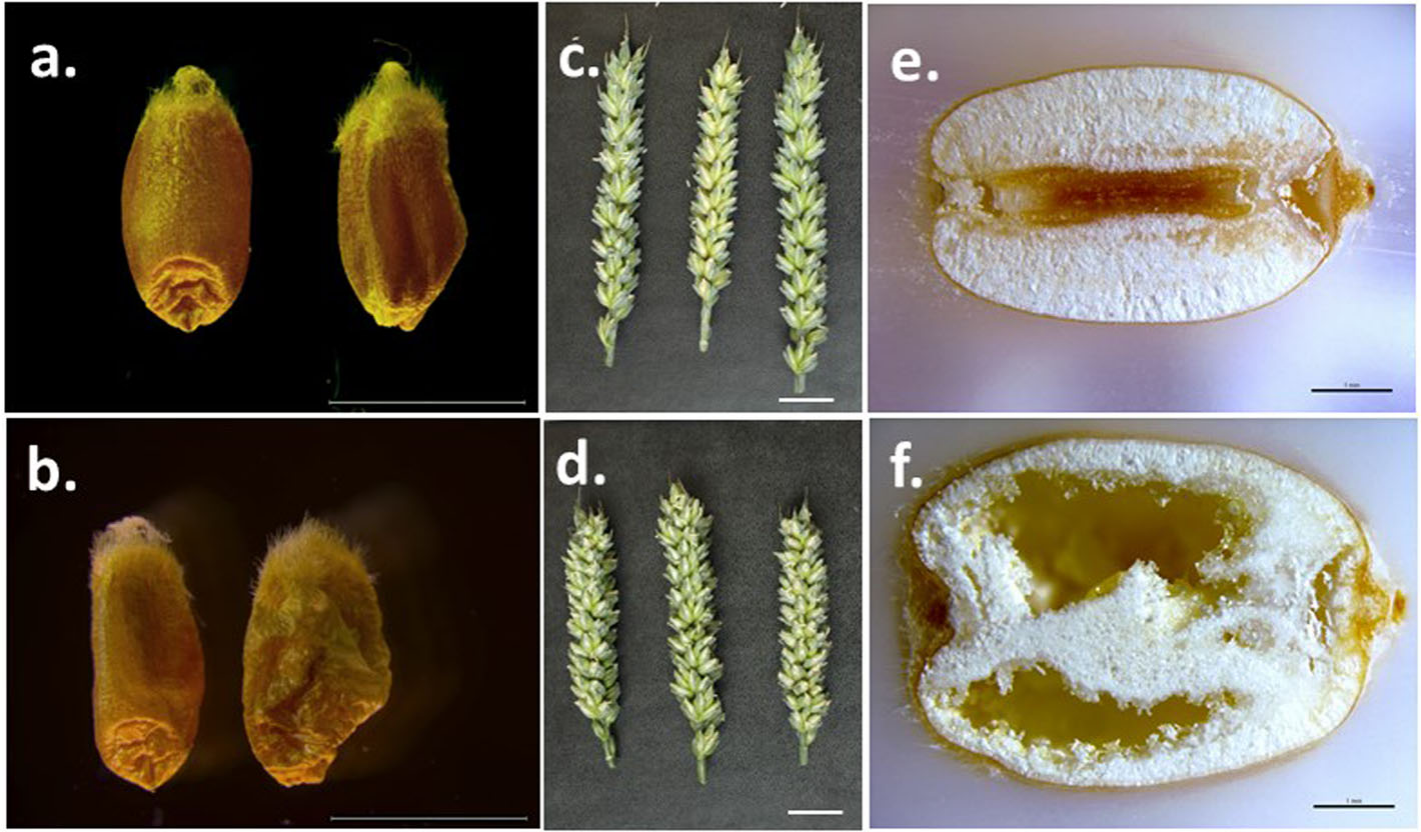
Light microscopy photos **a-b** showing the wrinkled and ‘collapsed’ phenotype of *AsWRI1*-expressing wheat grains at maturity. Photos **c-d** visualising the ‘swollen’ phenotype of grains on spikes at approximately 26 dpa and light microscopy photos **e-f** showing the interior cavity in *AsWRI1*-wheat grains **f** at this developmental stage (dark area of the grain in e is the crease). Upper row; control (null), lower row; homozygous line with multiple gene inserts. Scale bars are 10 mm **a-b**, 20 mm **c-d** and 1 mm **e-f**

Mature grains from homozygous *AsWRI1*-wheat lines with different numbers of gene inserts (one, two or twelve, Table S1) showed a wrinkled seed phenotype as compared to the corresponding azygous (null) segregants (Fig. S1), as illustrated for the line with twelve inserts in Fig. 1a-b. The caryopses at the later stage of development (26 days post anthesis (dpa)) had a swollen appearance (Fig. 1c-d) and a clear aqueous liquid seeped out when the caryopsis was cut with a scalpel. Light microscopy of caryopses revealed an interior with a much reduced volume of starchy endosperm and a cavity that, in the micrographs, appeared empty (Fig. 1e-f) but that contained the clear liquid described. However, the endosperm appeared to be intact around the periphery of the grains.

**Fig. 2:**
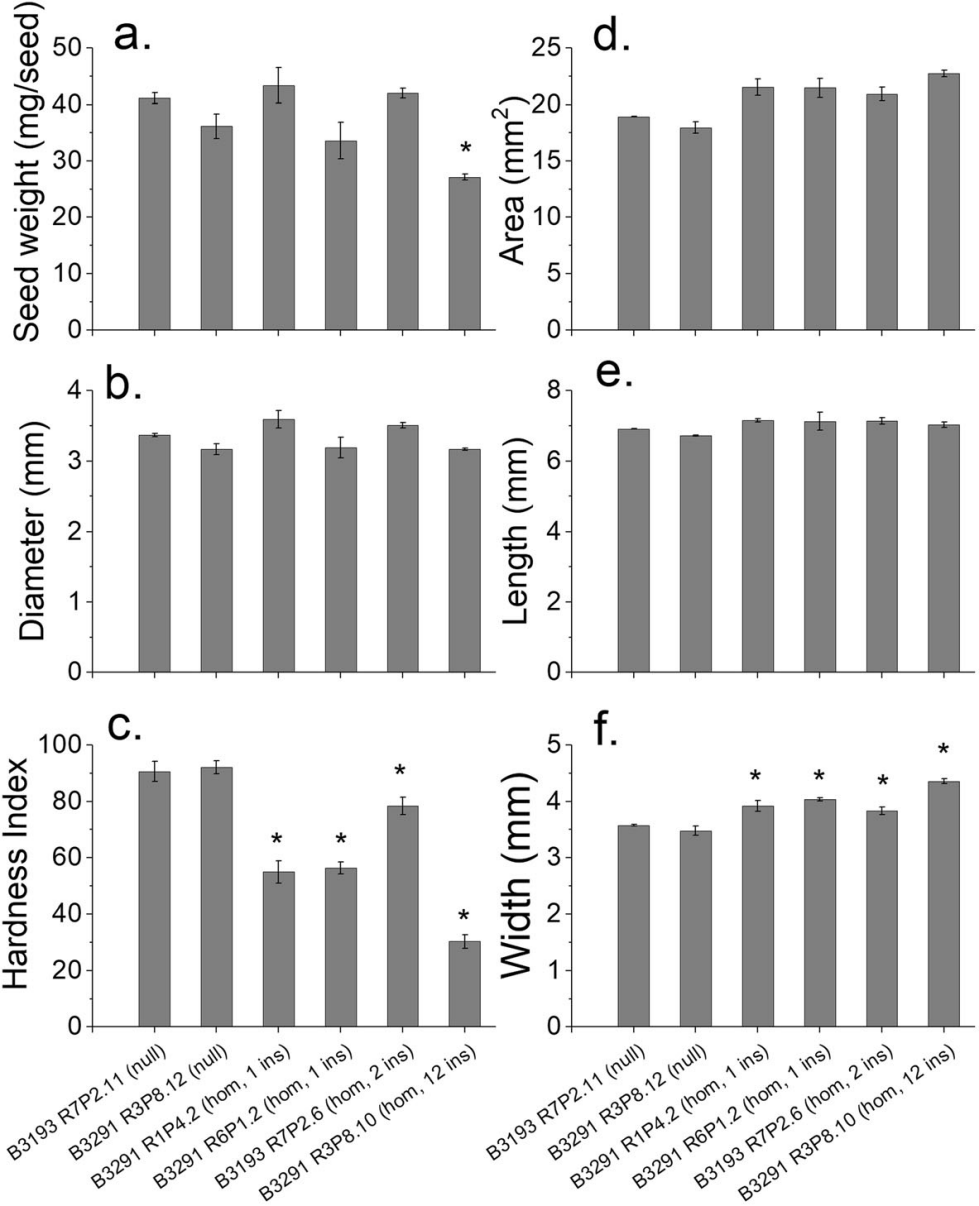
Grain weight **a**, diameter **b**, hardness **c**, area **d**, length **e** and width **f** in four homozygous *AsWRI1*-wheat lines of T4 generation (one, two or 12 inserts (ins)) and in null lines. Seed parameters were measured on mature grains using the Single Kernel Characterisation System (SKCS) **a-c** or MARVIN **d-f**. The results are shown as the mean ± standard error from three biological replicates, each consisting of 100 seeds. The asterisks indicate where the transgenic line is significantly different (*p* < 0.05, LSD) from null lines

The weights of mature grains of *AsWRI1*-wheat were reduced by 29% in the line with 12 inserts but were not affected in the other homozygous lines, as compared to the null lines (Fig. 2a). The diameters of grains were not significantly changed in the *AsWRI1*-wheat lines (Fig. 2b). The grain hardness index was greatly reduced, by 67% at most for the multiple insert line, in *AsWRI1*-wheat as compared to the control (Fig. 2c). Mature grains from the multiple insert line were also fragile and cracked easily. Other grain parameters measured were area, length and width among which only the latter showed significant increases for all *AsWRI1*-lines as compared to null lines (Fig. 2d-f). The germination rates did not differ from the null lines (Fig. S2).

**Fig. 3:**
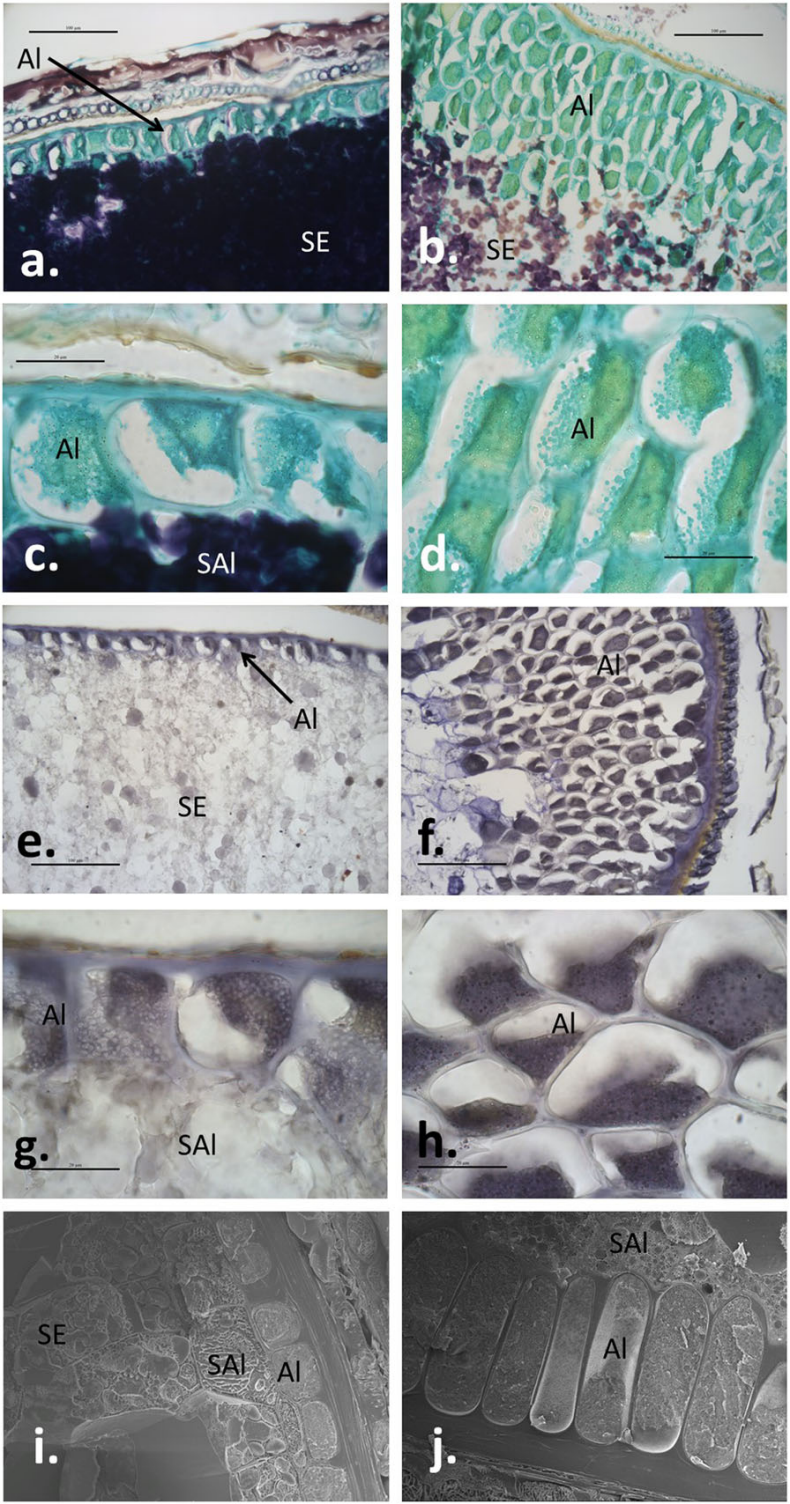
Structural analyses of fixed and sectioned wheat grains using light microscopy (**a-h**, 26 dpa) and scanning electron microscopy of freeze fractured grains (**i** and **j**; 21 and 28 dpa respectively). Control (on the left, **a, c, e, g, i**) and homozygous *AsWRI1*-wheat line with multiple insert (on the right, **b, d, f, h, j**). Grain sections stained with MAS staining proteins green and starch in dark purple **a-d** and Sudan black staining lipids in dark **e-h**. Al; aleurone cells, SE; starchy endosperm, SAl; subaleurone cells. Scale bars 100 μm **a, b, e, f**, 20 μm **c, d, g, h** and 50 μm **i, j**

Light microscopy of sectioned caryopses at 26 dpa revealed that the peripheral aleurone cells, stained green by Light Green due to their high protein content, were elongated and at some positions present in multiple layers in *AsWRI1*-wheat , as compared with the control where only a single layer of cuboid peripheral aleurone cells were present (Fig. 3a-d). Aleurone cells are usually oil-dense in cereals and Sudan black (a lipid-specific stain) confirmed their high lipid content (Fig. 3e-h). Scanning electron microscopy of freeze-fractured grains at late developmental stages confirmed the phenotype in the *AsWRI1*-wheat showing the elongated peripheral aleurone cells (Fig. 3i and j).

### Oat endosperm WRI1 induces oil accumulation in wheat endosperm

**Fig. 4:**
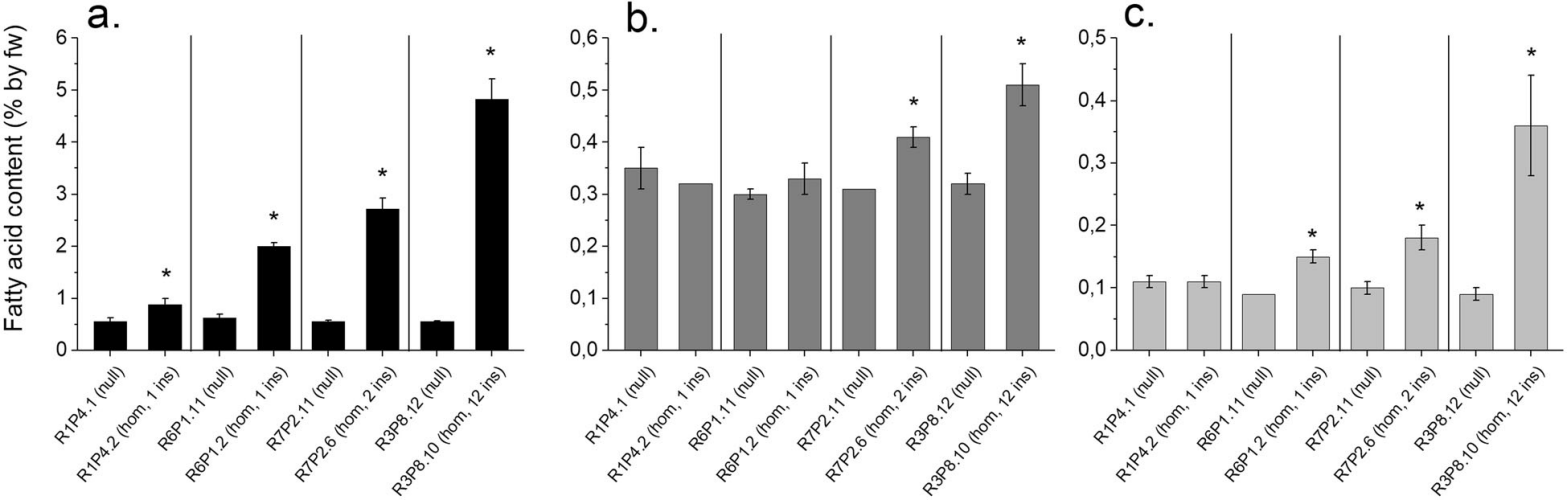
Lipid class content on fresh weight (fw) basis in endosperm (including starchy endosperm, aleurone cells, seed coat and pericarp) of mature grains of four homozygous lines (with one, two or twelve inserts (ins)) and their corresponding nulls (controls) of T2 generation. Triacylglycerol **a**, polar lipids **b**, and other remaining lipids **c**. The results are shown as the mean ± standard deviation from two biological replicates each consisting of three grains. Asterisks indicate significant differences as compared to corresponding nulls according to Fisher’s test with a significance threshold of *P* ≤ 0.05

To exclude the contribution of oil from the embryo (which accounts for about 3% of the whole wheat grain but typically is oil dense in all cereals), the embryo was excised from wheat grains leaving the remaining part which is hereafter called the endosperm (i.e. containing most of the starchy endosperm with associated parts of the aleurone, seed coat, and pericarp). The total fatty acid content of the endosperm of mature grains was significantly increased in 12 out of 16 homozygous *AsWRI1*-wheat lines as compared to the corresponding null segregants (Fig. S3). Analyses of the four lines with the highest total fatty acid content showed that this increase resulted mainly from increased amounts of TAG (Fig. 4a). The TAG content was much higher in the line with 12 inserted gene copies, as compared to the lines with only one or two. The contents of polar lipids and other lipids (i.e. mainly monoacyl- and diacyl- glycerols) were increased in two and three, of the homozygous lines, respectively (Fig. 4b and c), even though it can be noted that the absolute levels of these lipids were much lower as compared to TAG.

**Fig. 5:**
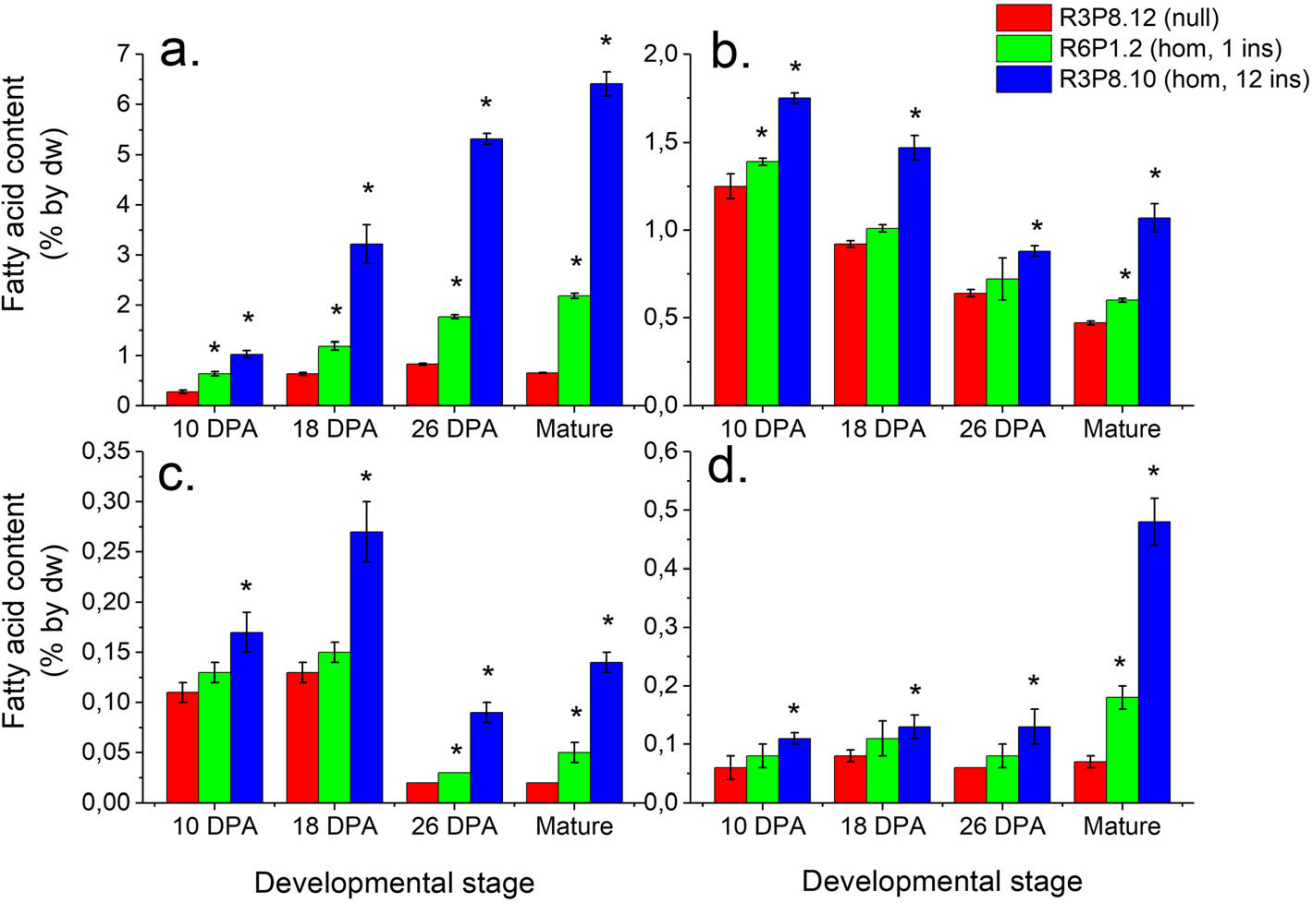
Lipid class content on dry weight (dw) basis in endosperm (including starchy endosperm, aleurone cells, seed coat and pericarp) of developing grains from selected homozygous (hom) lines (with one, two or 12 inserts (ins)) and control (null) from T3 generation. Triacylglycerol **a**, polar lipids **b**, free fatty acids **c**, other remaining lipids **d**. The results are shown as the mean ± standard deviation from three biological replicates. Asterisks indicate significant differences as compared to control according to Fisher’s test with a significance threshold of *P* ≤ 0.05

Analysis of two homozygous lines (with one and 12 inserted gene copies, hereafter named the single and multiple insert lines, respectively) during grain development revealed that TAG content was higher already at 10 dpa with larger differences at later stages, as compared to the null line (Fig. 5a). At grain maturity, the TAG content of the multiple insert line was more than nine-fold higher than of the null line (6.4% compared to 0.7% by dw). The polar lipid contents were higher in the multiple insert line all through grain development with a two-fold increase by maturity (Fig. 5b). Free fatty acids and other acyl lipids were present at up to 7-fold higher levels in the *AsWRI1*-wheat lines as compared to the null line (Fig. 5c-d). The greatest changes in the fatty acid profiles in the *AsWRI1*-wheat lines were increased proportions of oleic acid (18:1) and decreased proportions of linoleic acid (18:2) in polar lipids and free fatty acids (Fig. S4). Similar changes in fatty acid profile were observed for TAG but to a much smaller extent.

The amount of oil per grain (excluding the embryo) increased over five-fold, from approximately 0.3 mg TAG in the control to 1.6 mg in the multiple insert *AsWRI1*-wheat at maturity (calculated from data on seed weight and TAG content). This shows that the increased oil content in grains expressed on a % basis did not result from the reduced seed weight. However, the presence of multiple layers of highly elongated aleurone cells in *AsWRI1-*wheat raised the question of whether the increased TAG content in grains was a result from an increase in the aleurone cells, as opposed to an increase in the starchy endosperm cells which was expected due to the tissue specificity of the *HMW1Dx5* promoter which was used to drive *AsWRI1* [31]. The distribution of TAG in grains from the multiple insert and null lines at 26 dpa was therefore determined after dissection into embryo, starchy endosperm, and outer layers (including aleurone).

**Fig. 6:**
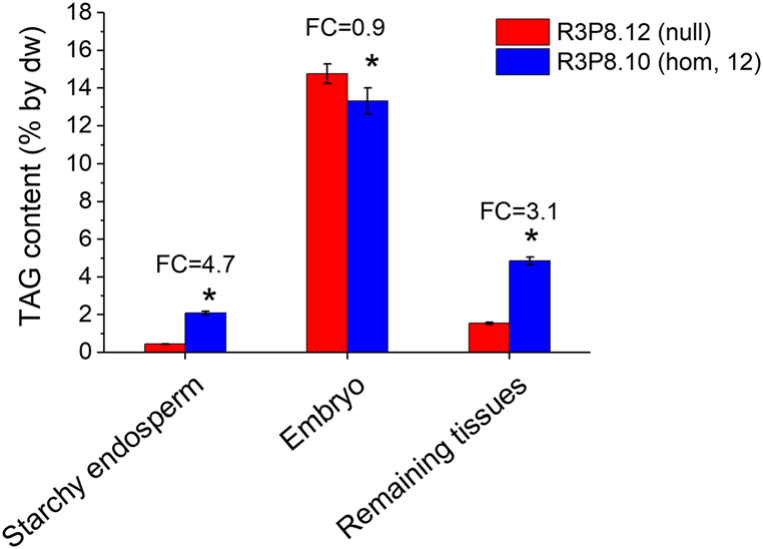
Triacylglycerol content on dry weight (dw) basis in different grain tissues dissected from *AsWRI1*-expressing wheat at approximately 26 dpa in the multiple insert homozygous (hom) line and control (null). The remaining tissues include the aleurone, seed coat, pericarp and in the homozygous line some starchy endosperm due to difficulties in dissection. Fold changes (FC) of differences in multiple insert line as compared to control are given for each tissue. The results are shown as the mean ± standard deviation from three biological replicates. Asterisks indicate significant differences as compared to control according to Fisher’s test with a significance threshold of *P* ≤ 0.05

The largest fold change in total TAG content was found in the starchy endosperm, increasing from 0.4% by dw in the null to 2.1% in the multiple insert line (Fig. 6). The starchy endosperm is the major storage tissue of the wheat grain and the more than 4-fold increase in oil content in this tissue is a drastic metabolic change. The TAG content was, as expected, highest in the embryo (14% by dw) with small differences between the lines. Due to the small contribution of the embryo to the total wheat grain [2] this oil does not contribute greatly to the total grain oil content. The ‘outer cell layers’ of the grain prepared by dissection included the pericarp, seed coat and aleurone, but also some starchy endosperm tissue (i.e. the outer cell layers were contaminated with starchy endosperm), particularly for the *AsWRI1*-wheat grains for which dissection was difficult. This tissue showed a 2.5-fold increase in TAG content between the null (1.8% by dw) and the multiple insert line (4.5% by dw). The aleurone cells usually comprise a small proportion of the wheat grain [2]. However, in addition to the increased amount of oil in the starchy endosperm, oil present in the aleurone cells contributed to the increased grain oil content of the *AsWRI1*-wheat, taking into account that the number of aleurone cell layers was increased, and that the starchy endosperm volume was much reduced. It can be noted that the clear liquid observed during seed development of *AsWRI1*-wheat did not contain any lipids as determined by visual inspection of total lipid extracts separated by thin layer chromatography.

### Reduction in starch and increase in sugars

**Fig. 7:**
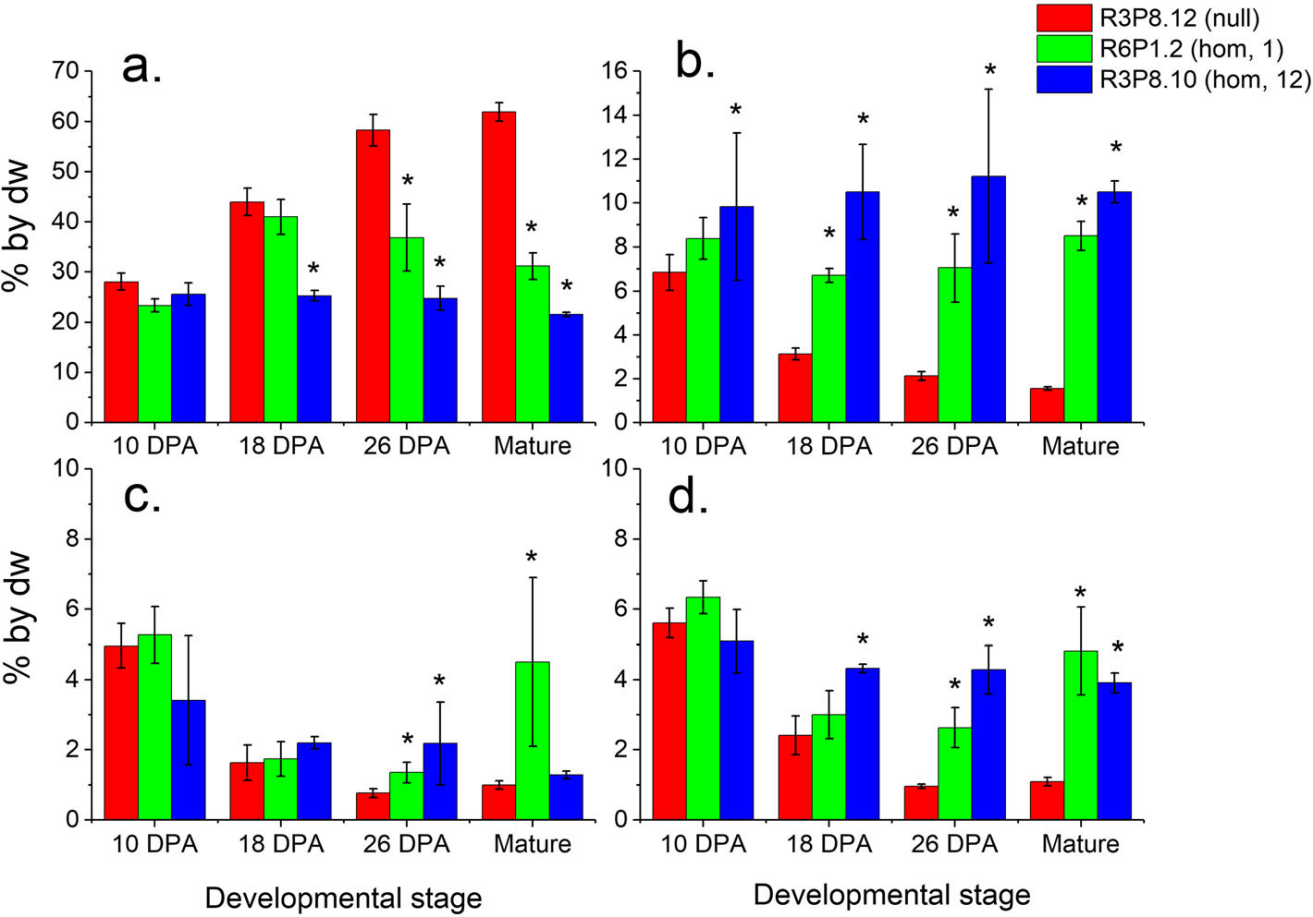
Starch **a**, sucrose **b**, glucose **c**, and fructose **d** content on dry weight (dw) basis in endosperm (including starchy endosperm, aleurone cells, seed coat and pericarp) of developing grains from selected homozygous (hom) *AsWRI1*-wheat lines (with one or 12 inserts) and control (null) from T3 generation. The results are shown as the mean ± standard deviation from three biological replicates. Asterisks indicate significant differences as compared to control according to Fisher’s test with a significance threshold of *P* ≤ 0.05

The starch content of the grain endosperms from homozygous *AsWRI1*-wheat lines was drastically reduced as compared to the null lines (Fig. 7a). This was already evident for the multiple insert line at 18 dpa, but was much more pronounced at later stages of development with the homozygous lines showing 50–63% reduction in starch content compared to the null line at maturity.

The sucrose content was instead greatly increased in grain endosperms of the *AsWRI1*-wheat lines compared to the control (Fig. 7b). This was apparent from 18 dpa onwards, and by maturity the homozygous lines contained up to 8–10% sucrose by dw in contrast to only 1.5% in the control. The glucose content of the grains did not vary greatly but an increase was observed in homozygous lines at 26 dpa as compared to the control (Fig. 7c). The content of fructose was higher in the *AsWRI1*-expressing grains, being approximately 4% by dw at maturity compared to 1% by dw in the null line (Fig. 7d).

To estimate how the carbon balance of wheat endosperm was affected by *AsWRI1* expression, we calculated total carbon amounts as starch equivalents in the endosperms of *AsWRI1*-wheat and control. The starch amount on a per endosperm basis decreased from 22 mg in the control to only 5 mg in the multiple insert *AsWRI1*-wheat at maturity. If all of the carbon in this starch loss (17 mg) was reallocated into oil synthesis in the endosperm it should give an increase of 6.8 mg oil (taking into account that the energy content of oil is approximately 2.5 times higher than that of starch on a weight basis). Nevertheless, the increase in oil in *AsWRI1*-wheat endosperm compared to the control was only 1.3 mg per grain. The increase in sugars (sucrose, fructose and glucose) of 2.5 mg per endosperm in the multiple insert *AsWRI1*-wheat explains some of the remaining carbon loss from starch, but there was still a difference of 6.9 mg per endosperm. Protein content did not differ significantly between lines (Fig. S5).

### Pulse-chased ^14^C-sucrose shows drastic changes in carbon allocation

To better understand how carbon allocation was affected by expression of *AsWRI1*, ^14^C pulse-chase labelling of seeds developing on detached spikes was performed using a similar in vitro system to those previously used to study oat and wheat [32–34]. The net accumulated ^14^C from fed sucrose in the endosperm of grains was approximately the same, about 50000 dpm/grain for the multiple insert line and the control (Fig. S6), showing that it was valid to compare the proportional distributions of ^14^C between different grain fractions during development. Since no reduction in net ^14^C label was observed during the chase period, it is unlikely that a significant proportion of the ^14^C label was lost by respiration during the studied time interval.

**Fig. 8:**
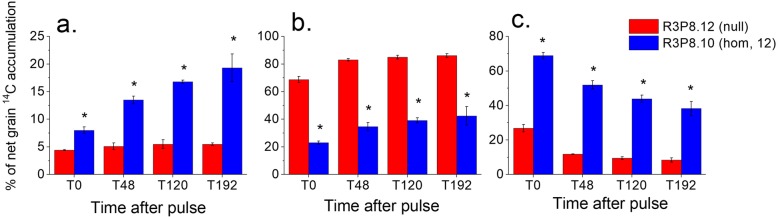
Distribution (%) of net accumulated ^14^C between different fractions of wheat grain endosperm (including starchy endosperm, aleurone cells, seed coat and pericarp) of the multiple insert line (hom, 12) and control (null) at different time points (T) 0–192 h after given ^14^C-sucrose pulse to detached spikes of wheat. Lipids **a**, starch/cellulose **b**, and water/methanol soluble compounds **c**. Proteins can be expected to be present in water/methanol fraction (soluble ones) and in the starch/cellulose fraction (non-soluble ones). The results are shown as the mean ± standard deviation from three biological replicates. Asterisks indicate significant differences as compared to control according to Fisher’s test with a significance threshold of *P* ≤ 0.05

A much higher proportion of the net accumulated ^14^C in the grain endosperm was recovered in the lipid fraction in the *AsWRI1*-wheat, increasing from 8% at 0 h to 20% at 192 h after the pulse, compared to only 5% in the control (Fig.8a). By contrast, a much lower proportion of ^14^C in the *AsWRI1*-wheat was allocated to the starch/cellulose fraction as compared to the null line; 23 and 69% respectively, at 0 h after the pulse, then increasing up to 42 and 86%, respectively, at 192 h (Fig. 8b). The highest proportion (69%) of ^14^C was recovered in the water/methanol soluble fraction in the *AsWRI1*-wheat at 0 h, compared with only 27% in the control line (Fig. 8c). Although it decreased over time, the water/methanol soluble fraction still accounted for 38% of the total^14^C in the *AsWRI1*-expressing line at 192 h, compared to only 8% in the control.

Analysis of ^14^C in different lipid classes showed that the largest difference was in TAG, with six-fold higher accumulation in the endosperm of *AsWRI1*-wheat grains as compared to the null at 0 h (Fig. S7a). While the net accumulation of ^14^C in TAG increased in *AsWRI1*-wheat over the whole pulse-chase period of 8 days, it only increased between the two first time points in the null line. A similar pattern was seen for the net accumulation of ^14^C in free fatty acids but the absolute levels were much lower (Fig. S7c). No large differences were observed in other types of lipids (Fig. S7b, d, e).

### Transcriptomics reveals increased fatty acid synthesis and decreased starch synthesis

**Fig. 9:**
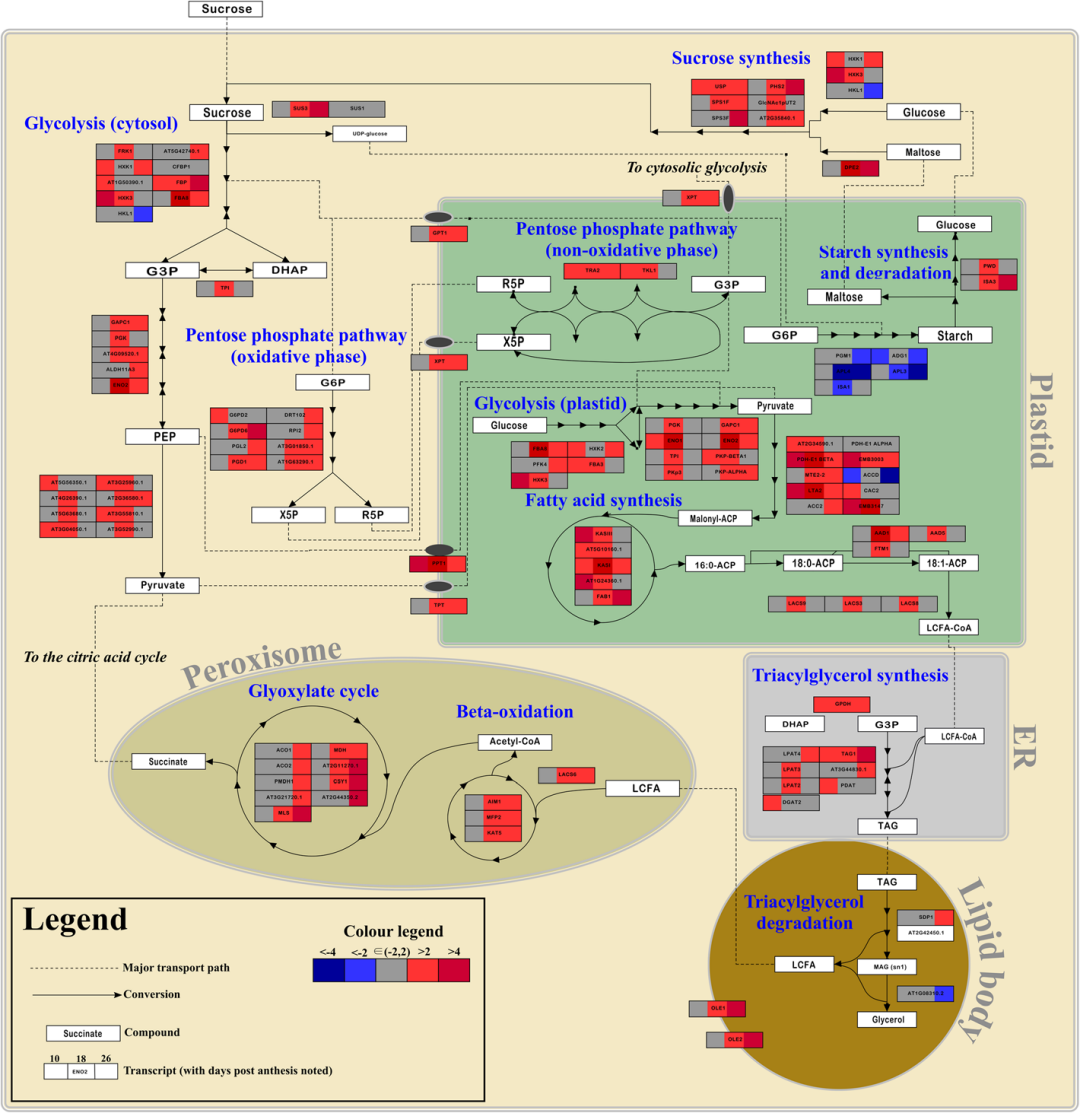
Heat map showing fold change between endosperm of *AsWRI1*-wheat (with multiple inserts) and control in log_2_ in transcript abundance of genes involved in the central carbon metabolism modeled from Arabidopsis. Only genes where a more than two fold change in transcript abundance in at least one of the time points (10, 18 or 26 days post anthesis) are shown. Gene symbols are based on TAIRs Gene Class Symbol Registry and when such is missing the AGI locus code is presented. Abbreviations: endoplasmic reticulum, ER; glyceraldehyde 3-phosphate, G3P; dihydroxyacetone phosphate, DHAP; 2-phosphoenolpyruvate, PEP; glucose 6-phosphate, G6P; xylulose 5-phosphate, X5P; ribose 5-phosphate, R5P; acyl carrier protein, ACP; coenzyme A, CoA; triacylglycerol, TAG; monoacylglycerol, MAG; long chain fatty acid, LCFA; uridine diphosphate, UDP. Results shown are the mean values from three biological replicates of endosperms (including starchy endosperm, aleurone cells, seed coat and pericarp) from the multiple insert *AsWRI1*-wheat line as compared to corresponding null (control)

As a complement to the morphological, analytical and biochemical studies, gene transcript levels were determined using high-throughput sequencing of mRNA from endosperm of developing grains of the multiple insert *AsWRI1*-wheat line and the null line. Reads were mapped to the TGACv1 wheat cDNA assembly and Arabidopsis protein sequences (AraPort v11) were used for annotation for metabolic mapping. *AsWRI1* was highly expressed at the earliest stage of analysis (10 dpa) with transcripts per kilobase million (TPM) values of approximately 5000 that gradually decreased to about 100 at 26 dpa. Genes were defined as up-regulated when fold changes ≥2 in the multiple insert line and down-regulated when fold changes ≤ − 2, as compared to the null line. Based on gene ontology, differentially expressed transcripts relating to central carbon and fatty acid metabolism (Table S2) were plotted using heat maps (Fig. 9).

Several genes involved in the cytosolic glycolysis pathway and de novo fatty acid synthesis, located in the plastid, showed increased transcript abundances in the *AsWRI1*-wheat line as compared to the control (such as the cytosolic *FRUCTOSE-1,6-BISPHOSPHATASE* (AT1G43670.1) and the plastid localised *PYRUVATE DEHYDROGENASE E1 BETA* (AT1G30120.1)). Associated with this was increased transcript abundance of *PHOSPHOENOLPYRUVATE/PHOSPHATE TRANSLOCATOR1* (AT5G33320.1), which is involved in transport of phosphoenolpyruvate, a major substrate for fatty acid synthesis, across the inner membrane of the chloroplast. Within the plastid, the transcript levels of several genes involved in the conversion of glucose 6-phosphate to ADP-glucose, which is a key precursor to starch, were down-regulated, such as *ADP-GLUCOSE PYROPHOSPHORYLASE LARGE SUBUNIT 4* (AT2G21590.1) and *ISOAMYLASE1* (AT2G39930). Two genes involved in the degradation of starch were up-regulated.

Only a few genes involved in TAG-biosynthesis showed increased transcript levels, most notably the *TRIACYLGLYCEROL BIOSYNTHESIS DEFECT 1* (AT2G19450.1). At the same time the *AsWRI1*-wheat grains showed increased expression of several *OLEOSINS* involved in the formation of oil bodies required for storage of TAG such as *OLEOSIN 1* (AT4G25140.1). Transcripts encoding proteins involved in TAG-degradation (SUGAR-DEPENDENT1, AT5G04040.1) as well as almost all of the genes encoding functions in the peroxisomal β-oxidation of fatty acids and the connected glyoxylate cycle were up-regulated. Most of the genes encoding functions involved in the pentose phosphate pathway showed increased transcript abundances in the *AsWRI1* line compared to the control line, e.g. *GLUCOSE-6-PHOSPHATE DEHYDROGENASE* (AT5G40760.1).

## Discussion

### Reallocation of carbon from starch to oil in wheat endosperm

This study shows that although oil accumulation can be initiated and greatly increased in the wheat endosperm by expressing the transcription factor WRINKLED1, it is not a simple redistribution of fixed carbon towards oil. Analytical, biochemical, microscopy, and transcriptome data showed drastic effects on storage metabolism induced by the oat endosperm *WRI1* (*AsWRI1*) when over-expressed using the starchy endosperm-specific promoter *HMW1Dx5* in wheat grains. *AsWRI1* has been shown to be highly expressed in the oat starchy endosperm tissue [20] which, uniquely among cereals, accumulates substantial amounts of oil. *AsWRI1* has also been shown to induce oil accumulation when transiently expressed in *Nicotiana benthamiana* (Domin) leaves [21]. At maturity, the endosperm of *AsWRI1*-wheat had up to nine-fold increase in TAG (from 0.7 to 6.4% oil by dw) with a concomitant greatly reduced accumulation of carbon in starch (from 62 to 22% by dw), and an increased proportion of carbon in sucrose (from 1.5 to 10% by dw). These changes were presumably responsible for the wrinkled seed phenotype observed, as in the classic wrinkled pea which has a mutation in starch synthesis [35]. The size and the oil content of the embryo did not differ between the *AsWRI1* and control lines which contrasts with high-oil maize varieties [10, 36].

The approach to increase maize seed oil by expression of a *WRI1* homolog from the maize embryo in the starchy endosperm did not result in any change in carbon allocation into oil in this tissue [30]. By contrast, *AsWRI1* expression in the starchy endosperm of wheat increased the carbon allocation into oil significantly with the TAG level (6% by dw) being similar to that commonly found in grains of oat varieties which contain 3–18% oil by dw. Although the seed weight was reduced significantly in the line with multiple inserts of *AsWRI1*, mainly due to the reduction in starchy endosperm, the total amount of oil on a per grain basis at maturity was increased five-fold which shows an overall increased capacity for oil synthesis. While not specifically expressed in the seed, the constitutive expression from the maize ubiquitin promoter of a *WRI1* homolog from *Brachypodium distachyon* in the same species increased the oil content of the grain endosperm tissue [37], but the level was much lower compared to those reported here for wheat. The much stronger effect on storage metabolism observed in *AsWRI1*-wheat grain endosperm may be explained by the use of the strong starchy endosperm-specific *HMW1Dx5* promoter [31] as compared to the constitutively expressed maize ubiquitin promoter [38].

Although *AsWRI1* expression was sufficient to increase the total amount of oil in the endosperm by five-fold on a per grain basis, the seed weight was reduced by 29% at maturity. Partitioning of carbon into oil instead of starch could be expected to result in at least some decrease in seed weight because the energy density of oil on a weight basis is approximately 2.5 times higher than that of starch. However, the increased amount of oil in the endosperm of mature grains of *AsWRI1*-wheat did not correspond to the carbon loss in starch as compared to the control, and the observed increase in sugar did not account for the difference that remained. In fact, the endosperm in mature grain of the multiple insert line had a 45% reduction in total carbon per grain (estimated as carbohydrate equivalents present in oil, starch, and sugars) as compared to the control. On the other hand, in vitro pulse-chase analysis using ^14^C sucrose showed that the net carbon accumulation per endosperm did not differ between the *AsWRI1*-wheat and the control at the mid-stage of grain development. This may imply that the carbon sink strength of the grains expressing *AsWRI1* was severely reduced after the mid-stage of grain development. It is possible that the transport of photosynthate into grains is negatively affected by the increased sucrose content in grains which reduces the sugar gradient between source and sink.

Light microscopy of grains at a late developmental stage showed that the starchy endosperm cells were intact around the periphery of the grains expressing *AsWRI1*, but that the interior of the tissue lacked a cellular structure and instead contained an aqueous liquid, in contrast to the continuity of the cellular structure throughout the grain in control plants. Furthermore, ^14^C-sucrose labelling experiments indicated that carbon was apparently trapped in the water/methanol soluble fraction of *AsWRI1*-wheat endosperms, and nuclear magnetic resonance (NMR) analysis showed that the endosperm contained greatly increased levels of soluble sugars such as sucrose, as compared to control. It is therefore likely that the allocation of carbon from sugars into starch was severely reduced, or starch degradation was increased, in the *AsWRI1* lines, leading to the increased accumulation of sugars in addition to increased synthesis of lipids. It can be speculated that the early stages of endosperm development [39] might function normally in the wheat expressing *AsWRI1*, but the effect on grain physiology of the much decreased allocation into starch synthesis becomes more severe over time which could possibly lead to the concomitant build-up of sucrose. This increased effect on metabolism observed during grain development is consistent with the timing of the promoter used for expression of *AsWRI1*, with expression from about 8–10 dpa [31, 40].

### Effects on aleurone cells

The accumulation of nutrients in wheat show a gradient with different grain tissues having different nutritional compositions which have implications for milling and processing [41, 42]. The outer layer(s) of endosperm cells in cereals form the aleurone layer and differ from the starchy endosperm cells in having thick walls and high contents of non-gluten proteins, lipids, vitamins and minerals. In oat grains the storage lipids accumulate throughout the starchy endosperm but are more abundant in the outer endosperm [4, 5]. Although the highest fold change in TAG content in the *AsWRI1*-wheat grains at a late stage of development as compared to control was found in the starchy endosperm fraction, the fold-change in TAG content of the outer cell layers (including the aleurone, seed coat and pericarp) also contributed to the overall increased oil content of the grain. Microscopy of the *AsWRI1*-wheat grains showed morphological changes in the peripheral aleurone cells, which were elongated and present in multiple layers at some positions. This effect was striking because in most cereal species, including wheat and oat, the peripheral aleurone layer comprises only a single layer of cells which are typically cuboid in shape. Of the cultivated cereals only barley (*Hordeum vulgare* L.) has multiple layers of aleurone cells (up to four) [43–45] but the developmental fate of endosperm cells into aleurone cells seem to show plasticity also in other cereals as shown by studies on maize and rice mutants [46, 47]. The changes observed in the *AsWRI1*-wheat aleurone cells were surprising taking into account that expression of *AsWRI1* was driven from the high-molecular weight glutenin promoter which is known to be active in wheat grain starchy endosperm but not in aleurone cells [31]. However, in addition to functioning as carbon and energy sources sugar concentrations also affect sink strength in general and have a role in signalling to control metabolism, growth and development in plants [48, 49]. It can be speculated that the high sucrose content in the endosperm of *AsWRI1*-wheat grains (10% by dw) could have an indirect effect on the development of and storage accumulation in the aleurone cells which surround the starchy endosperm cells in which *AsWRI1* is expressed.

### Transcriptomic transitions and metabolic implications

Transcriptome analyses revealed that genes involved in starch biosynthesis were down-regulated while genes in starch degradation were up-regulated in the *AsWRI1*-expressing wheat endosperm which is similar to the pattern observed upon oil induction by *AsWRI1* and other *WRI1* homologs in tobacco leaf [21]. Down-regulation of genes in starch synthesis was also observed in *WRI1*-expressing potato tubers [29]. This indicates that an important indirect function of WRI1 may be to divert carbon from starch by transcriptionally reducing starch synthesis potential accompanied by starch degradation. Consequently, a high proportion of the sucrose transported through the crease into the developing endosperm [50] probably accumulates rather than being converted into starch which may partly explain the high sucrose content observed in *AsWRI1*-wheat endosperm. More detailed studies of the *AsWRI1*-lines with single or double inserts, which did not show reduced seed weights and with smaller reductions in starch content as compared to the control, can provide further insight to the relations between transcript abundance and effects on reserve partitioning in order to minimise undesired phenotypic effects.

The results of our transcriptome analyses confirmed the effects of WRI1 on the transcriptional regulation of several genes encoding functions in cytosolic glycolysis, de novo plastid fatty acid synthesis and also the transport of triose-phosphates across the plastid membrane that have previously been reported for dicot seeds, leaf tissue and potato tuber [21, 25, 29]. Although a few genes encoding functions in TAG assembly pathways were found to be up-regulated in *AsWRI1*-wheat grains, our results agreed with previously observed effects of WRI1 on the transcriptional regulation of fatty acid synthesis, rather than of TAG biosynthesis [15, 16, 51, 52]. Oleosins (oil body proteins) play a structural role in oil bodies and oleosin-encoding genes were up-regulated in *AsWRI1*-wheat grain endosperm. This was also observed in *WRI1*-expressing potato tubers [29] but not in *WRI1*-expressing leaves [21].

Accumulation of free fatty acids which are highly hydrophobic is known to have cytotoxic effects in microalgae and plants [53–56]. Fatty acids are usually bound to proteins, both during de novo synthesis in the plastid as well as during cellular distribution of acyl chains (for example into lipid assembly) thereby reducing their cytotoxicity [23, 57]. But during an overload of fatty acids such proteins could probably be limiting which could cause induction of fatty acid degradation. Whereas the TAG that accumulated with ectopic *WRI1* expression in leaves of both monocot and dicot plant species was turned over [21, 37, 58], the TAG that accumulated when *WRI1* was expressed in potato tubers was not [29]. Transcriptome analysis indicated that TAG degradation and especially the peroxisome degradation of fatty acids were up-regulated in the *AsWRI1*-wheat endosperm. Free fatty acids were also shown by chemical and ^14^C-labelling analyses to increase in *AsWRI1*-wheat endosperm, which could result from both TAG degradation and from the highly induced de novo fatty acid synthesis as shown by the transcriptome analysis. Increased fatty acid synthesis concurrent with degradation would indicate that fatty acids are futile cycled in the *AsWRI1*-wheat. Altogether this may imply that not only TAG turnover and fatty acid synthesis degradation but also a non-sufficient TAG assembly (which cannot handle the increased availability of building blocks through increased de novo fatty acid synthesis) could be limiting factors for further increases in the *AsWRI1*-wheat endosperm. Furthermore, the futile cycling of fatty acids could potentially lead to reduced carbon import into grain due to an increased recycling of carbon molecules within the grain.

### Further increases of oil accumulation in wheat endosperm

Altogether our data indicates that to increase the oil content in wheat endosperm to a level that is relevant for exploitation without having negative effects on seed weight, expression of *AsWRI1* is not enough. In fact, the effects of expressing a transcription factor known to allocate carbon into oil can differ depending on the metabolic context, notably whether the plant tissue has a source or sink character. Co-expression of genes encoding different functions in TAG production (i.e. *WRI1*, *DIACYLGLYCEROL ACYLTRANSFERASE1* and *OLEOSIN*) using the push-pull-protect approach induced large changes in carbon metabolism in tobacco leaves resulting in an oil content of 15% by dw [59]. Our results suggest that even if the TAG assembly pathway is considered not to limit TAG accumulation in some plant storage tissues [15, 16, 51, 52], it was suggested to be limiting in oilseed rape [60] and it may also limit oil accumulation in the cereal starchy endosperm. TAG assembly could therefore be a target (i.e. acyltransferases) in order to increase the allocation of carbon into oil and reduce futile cycling of carbon in the *AsWRI1*-wheat endosperm. Even though genes encoding oleosins were up-regulated in the *AsWRI1*-wheat endosperm, further increases in oleosins in this tissue could protect the newly formed TAG from degradation since TAG turnover was indicated to be a constraint for increased oil content. Suppression of lipases increased the TAG content in Arabidopsis and tobacco leaves [58, 61] and suppression of a lipoxygenase gave increased fatty acid content in wheat grains [62], suggesting that these enzymes could also be targeted to reduce the turnover of TAG to achieve increased oil content in the cereal endosperm.

## Conclusions

Our data for the first time show the changing metabolic patterns occurring upon expression of oat endosperm *WRI1* in the wheat endosperm, which is one of the world’s most important sources for food and feed. Future studies of oat and wheat may elucidate the mechanisms regulating the developmental and metabolic pathways required to allow the accumulation of both oil and starch in the cereal endosperm cells without diverting a high share of carbon into soluble sugars and causing undesired phenotypic effects.

## Methods

### Overexpression construct and transformation

An overexpression construct of the endosperm *WRI*1 from *Avena sativa* (1338 bp) [21] under the control of the starchy endosperm-specific *HMW1Dx5* promoter [31] and *HMW* and 35S terminator was created using a *Sal1*/*Xba*1 cloning strategy. The fragment was generated by PCR (Phusion *Taq* polymerase, Fisher Scientific, Leicestershire, United Kingdom), using PCR primers *WRI*1OEF: CATGTCGACATGAAGAGATCCCCGCC and *WRI*1OER: CATTCTAGATCAATTACACACAGTGA.

Sequencing of the constructs was carried out using the BigDye Terminator Version 3.1 Cycle Sequencing Kit (Applied Biosystems, Life Technologies Ltd., Paisley, UK), with construct-specific primers Rab1: CACAACACCGAGCACCACAAACT and DX5R2: CATTATTACTGGGCTTTACTC (Department of Biochemistry, University of Oxford, Oxford, UK). Transformation was done by particle bombardment (PDS1000; Bio-Rad, Hercules, USA) of immature scutella (10–14 d.p.a) of cv. Cadenza, co-bombarded with the pAHC20 plasmid, containing the selectable marker gene *bar* driven by the constitutive ubiquitin promoter from maize [38], as described by Sparks & Jones [63]. The transformation efficiency was 1.7%.

Genomic DNA was extracted from leaves using a Wizard kit (Promega Ltd., Hampshire, UK). Presence of the transgenes was confirmed by PCR using the Rab1 and *WRI*1OER primers in 25 μl reactions using a 1.1X ReddyMix™PCR Master Mix (1.5 mM MgCl_2_) from Thermo Scientific (ABgene House, Surrey, UK), ~ 200 ng DNA and 0.8 mM of each primer. Cycling conditions: 96 °C (5 min), 96 °C (32 × 30 s), 58 °C (30 sec), 72 °C (1.5 min) and 72 °C (10 min). PCR products were analysed on 1.0% (w/v) agarose gels, stained with ethidium bromide and visualised by UV light. Zygosity analysis of T1 plant material was done as described by Nemeth et al. [64] to identify transgenic lines with segregation patterns consistent with a single insertion locus, which identified homozygous lines with corresponding azygous nulls (controls) among segregants (Table S1, R, replicate number; P, plant number). It can be noted that six individual lines were further analysed which is important if taking into account the hexaploid nature of wheat.

### Plant growth and sampling conditions

Homozygous and azygous (null) segregants (T2) descended from the same original overexpression transformants (T1) were grown in glasshouse (Rothamsted Research, UK) with 18 °C-20 °C (day), 14 °C-16 °C (night) with a 16 h photoperiod provided by natural light supplemented with Son-T 400 W sodium lamps (Osram, Ltd., Munich, Germany) giving 400–1000 μmol m^− 2^ s^− 1^ photosynthetically active radiation. Developing T3 seeds were generated from plants grown in growth chambers (Biotron SLU-Alnarp, Sweden) under fluorescent light (250 μmol m^− 2^ s^− 1^) at 16 h photoperiod, 21 °C/18 °C (day/night), at 70% humidity (detached wheat spikes were also incubated in this chamber). Spikes were tagged by anthesis and grains harvested at specific developmental stages. The embryo was cut off from grains leaving the rest called the ‘endosperm’ (including starchy endosperm, aleurone cells, seed coat and pericarp). One biological replicate refers to one individual plant. Samples were snap freezed into N_2_ (l) and saved in − 80 °C. Plant tissues were ground in steel containers chilled in N_2_ (l) using a mixer mill (Retsch, Haan, Germany) at 30 Hz (15 s, 12 mm steel bead), saved in − 80 °C and freeze-dried prior to metabolite analyses.

### Grain analyses

Grain measurements (area, length and width) were determined using a Marvin Digital Seed Analyser using 100 grains from three biological replicates (CGTA Sensorik GmbH, Neubrandenburg, Germany). Hardness and individual seed weight was determined using the Perten Model Single Kernel Characterisation System 4100 (Perten Instruments, Calibre Control International Ltd., Warrington, UK). Twenty seeds from three biological replicates were used in germination tests. Seeds were washed in 70% Ethanol (10 min) followed by 20% hypochlorite solution (60 min). For the multiple insert line 20% hypochlorite was too harsh (seeds were fragile) and was reduced to 10%. Seeds were washed in sterile distilled water and placed in 90 mm Petri dishes with wet Whatman No. 1 filter paper, sealed with Parafilm and placed at 4 °C (2 days) to synchronise germination. The plates were incubated at room temperature (20-21 °C) and germination scored after 5 days as the presence of the radicle and hypocotyl (Fig. S2).

### Microscopy analyses

Grains at 26 dpa were split and put in fixative (2% (w/v) paraformaldehyde, 2.5% (v/v) glutaraldehyde in 0.1 M Sørensen phosphate buffer, pH 7.2) and incubated with agitation o/n. Samples were washed 3 × 15 min with buffer, then dehydrated using a series of ethanol (3 h each). Samples were cleared using a series of ethanol:xylene (12 h each) and infiltrated with Histowax Special (Histolab Products AB, Göteborg, Sweden) by incubating at 55 °C (3 × 24 h) before casted in molds. 12 μm sections were cut from blocks using a microtome and mounted onto glass slides (SuperFrost Plus, Menzel, Fisher Scientific, Waltham, USA). Paraffin was removed by sequential washes in xylene and EtOH (5 min each) at room temperature. Slides were stained at room temperature with 1) Light Green and Lugol’s solution and 2) Sudan Black [65] and mounted using Pertex Mounting solution. Stained sections were analysed using a light microscope (Leica, DFC 450C camera). For scanning electron microscopy, wheat grains were scored using a razor blade to aid fracturing and mounted on aluminium stubs, modified by drilling a hole to hold the grain, using a 50:50 mix of OCT compound (Sakura FineTek) and colloidal graphite (TAAB). The grains were plunge frozen in N_2_ (l) and transferred under vacuum to the GATAN ALTO 2500 cryo chamber maintained at − 180 °C. Samples were freeze fractured, etched at − 95 °C for 2 min and coated with a thin layer of Au-Pd. Samples were transferred to the cold stage of the JEOL 6700F scanning electron microscope, maintained at − 130 °C and imaged at 5 kV.

### Lipid analyses

Lipids from plant tissues were extracted according to a modified method of Bligh and Dyer [66] using 0.15 M acetic acid instead of water. Aliquots of total lipid extracts were either evaporated under N_2_ (g) followed by methylation yielding fatty acid methyl esters (FAMEs), or first separated into the different lipid classes using thin layer chromatography which were scraped off from plate followed by methylation. Methylation of lipids was done in 2% H_2_SO_4_ in MeOH. FAME identification and quantification were done using gas chromatography. For details of lipid analyses see Grimberg et al. [67]. It can be noted that the extraction method of Bligh and Dyer has previously been shown to give the highest extraction yields of total non-starch lipids (such as triacylglycerol, which is the major target lipid of our study) from wheat flour as compared to other extraction methods [68, 69]. Duplicate samples á 3 grains from each batch of T2 grains was used (Fig. S3 and Fig. 4), and lipid extracts corresponding to 10 mg dw from three biological replicates were separated on TLC (Fig. 5).

### Starch, sugar and protein analyses

Starch was determined on freeze-dried endosperm flour (50 mg) using the Total Starch Determination Kit (Megazyme, Wicklow, Ireland), with soluble sugars first removed with 80% aqueous EtOH.

Sugars were quantified using ^1^H-NMR spectroscopy as described previously [70]. In brief, freeze dried flour samples (15 mg) were extracted using 80:20 D_2_O:CD_3_OD containing 0.01% w/v d_4_-trimethylsilylpropionate as internal standard. ^1^H-NMR spectra were acquired at 300 K using an Avance spectrometer (Bruker, Coventry, UK) operating at 600.0528 MHz with a selective inverse probe. Spectra were collected using a water suppression pulse sequence with a 90° pulse and a relaxation delay of 5 s (128 scans of 64,000 data points with a width of 7309.99 Hz). Spectra were Fourier-transformed and ^1^H chemical shifts were referenced to TSP-d4 at δ 0.00. ^1^H NMR spectra were reduced using AMIX (Bruker BioSpin) to ASCII files containing integrated regions of equal width (0.001 ppm). Spectral intensities were scaled to the TSP-d4 region (from δ 0.05 to − 0.05). Carbohydrates were quantified against the known concentration of the internal standard using characteristic peaks from a library of authentic standards.

Amounts of 4 ± 0.2 mg of freeze dried seed flour from endosperm of grains were weighed into tin capsules (Mettler Toledo XP6) which were folded and analysed with an elementar analyzer (Flash 2000, Thermo Scientific). Produced NO_x_ was reduced to N_2_ (g) in a reduction column, separated from CO_2_ on a molecular sieve packed gas chromatography column and analyzed with a thermal conductivity detector. Data was analyzed using Eager Xperience software (ver 1.2, Thermo Scientific) to determine total N content. Total protein content was estimated as total Nx6.25.

### ^14^C pulse-chase experiment

Wheat spikes were labelled at anthesis and detached from plants at 14 dpa. Stems were surface sterilised in 70% EtOH (1 min), 1.0% chlorine (10 min) and rinsed in sterile water. The stem length was reduced to 7 cm by using a scalpel under water to eliminate cavitation. Spikes were transferred to plastic bottles with 10 mL sterile nutrient medium containing 1.47 g·L^− 1^ Murashige & Skoog media, 0.5 g·L^− 1^ 2[N-morpholino] ethane suplhonic acid, 0.5 g·L^− 1^ glutamin, and 40 g·L^− 1^ sucrose (Duchefa, Harlem, The Netherlands). D-[U-^14^C]-sucrose (PerkinElmer, Massachusetts, USA) was included in the medium in a concentration of 5700 dpm·nmol^− 1^ during the first 48 h. This ^14^C pulse was chased in spikes developing in medium without ^14^C-labelled substrate. Nutrient medium was changed and fresh cuts of stems were made every second day. Six grains were sampled from mid part of spikes directly after the given pulse (0 h) and up to 192 h. Grains were frozen in N_2_ (l) before cutting off embryo to harvest endosperms into plastic tubes further snap frozen in N_2_ (l) and saved in − 80 °C. Determination of ^14^C-label in different endosperm fractions was done as described previously [33] except that heptane:diethylether:acetic acid (70:30:1 volume) was used during separation of lipid classes.

### RNA-sequencing

Total RNA was extracted from grain endosperms ground in N_2_ (l) and stored at − 80 °C using PureLink Plant RNA Reagent (Ambion, Foster City, USA). Total RNA was DNase treated (TurboDNase, Ambion), the integrity and concentration measured using Experion RNA StdSens analysis kit (BioRad, Hercules, USA) and diluted to 60–340 ng·μL. Samples were exposed to paired-end Illumina HiSeq2500 sequencing with v4 chemistry (SNP&SEQ Technology Platform, National Genomics Infrastructure, Sweden). Libraries were prepared with TrueSeq stranded mRNA library kit with polyA selection (Illumina). RNA sequence data was processed using CLC Genomics Workbench v9.5.2. Sequences were trimmed according to standard settings and mapped to the wheat reference transcriptome TGACv1 (ensemble genomes [71]) according to standard settings. The wheat reference transcriptome was annotated against an Arabidopsis protein database (AraPort v11) using CLC Genomics Workbench v9.5.2 (blastx, E-values cut-off ≥0.01). Expression levels of genes are given as TPM (Transcripts Per kilobase Million). Differential expression of genes was calculated as the fold-change between the TPM in one biological replicate of *AsWRI1*-wheat and the mean of TPM in three biological replicates of the control. Gene expression data was visualized using PathVisio 3.3.0 [72, 73].

### Statistical analyses

Metabolite data was analysed using one-way analysis of variance (ANOVA) followed by Fisher’s test (*p* < 0.05, LSD) to compare means of treatment groups (Minitab 16). Fisher’s test was used because only predefined pair-wise comparisons within data sets (between transgenic lines and control, for each developmental stage or tissue) were of interest. Transformation of data using the natural logarithm was done prior to statistical tests when needed to stabilize variance and to obtain the approximate Normal distribution of residuals required for statistical inference.

Grain parameter data (weight, diameter, hardness, area, width and length) was analysed with ANOVA (two null lines for comparison to four homozygous lines, lines were nested in type for ANOVA). No transformation of data was required, assessment of residuals following analysis revealing broad confirmation to the assumptions of the analysis. Appropriate means were compared using the standard error of the difference (SED), thus invoking the least significant difference (LSD) at 5% level. Comparison of transgenic R3P8.10 to null R3P8.12, and of transgenic R7P2.6 to null R7P2.11 was made using the LSD. A generalized linear model assuming a Binomial distribution was used for the germination data with lines nested in type but noting that some over-dispersion (variance above and beyond that expected for the Binomial) was accounted for by an F-test rather than default Chi-squared. The Genstat (18th edition, VSN International Ltd., Hemel Hempstead, UK) statistics package was used.

## Supplementary information

**Additional file 1: Table S1 (excel file).** Zygosity tests of leaves from wheat lines transformed with oat *WRI1* (*AsWRI1*). Plants developed from segregating seeds. Bombardment codes (B = Bombardment Number; R = Replicate Number and P = Plant Number).

**Additional file 2: Table S2 (excel file).** List of differentially expressed genes in *AsWRI1*-wheat (multiple insert line) endosperm (including starchy endosperm, aleurone cells, seed coat and pericarp) as compared to in control presented in Fig. 9 at time points 10, 18 and 26 days post anthesis (dpa). The results are shown from three biological replicates. Values are fold change (FC) of transcripts per kilobase million (TPM) compared to control. The closest identified homolog in Arabidopsis is given as well as the Arabidopsis gene symbol and proposed enzymatic role. All Arabidopsis data were retrieved from TAIR (https://www.arabidopsis.org/index.jsp).

**Additional file 3: Figure S1.** Photos show the wrinkled seed phenotype of homozygous *AsWRI1*-wheat lines (upper row in each image) of T2 generation as compared to in their corresponding nulls (bottom row in each image) coming from six individual transformation events (a-f). Number of gene inserts (ins) are given in parentheses.

**Additional file 4: Figure S2.** Germination tests of *AsWRI1*-wheat grains in % of total grains. The results are shown as the mean ± standard error from three biological replicates, each consisting of 20 seeds. The generalized linear model showed that there were no real differences in the proportion of germination across the types (*p* = 0.110, F-test) or between the lines having accounted for type (*p* = 0.427, F-test). Number of gene inserts (ins) are given in parentheses. Hom, homozygous.

**Additional file 5: Figure S3.** Fatty acid content of total lipids on fresh weight (fw) basis in the endosperm (including starchy endosperm, aleurone cells, seed coat and pericarp) of mature *AsWRI1*-wheat grains from 16 homozygous (hom) lines (coming from six individual transformation events) and their corresponding nulls (controls; Ctrl) from T2 generation. The results are shown as the mean ± standard deviation from duplicate samples (á 3 grains) from seed batches from each line. Ins, gene inserts. Asterisks indicate significant differences as compared to control according to Fisher’s test with a significance threshold of *P* ≤ 0.05.

**Additional file 6: Figure S4.** Fatty acid profiles of different lipid classes in endosperm (including starchy endosperm, aleurone cells, seed coat and pericarp) of mature *AsWRI1*-wheat grains from selected homozygous (hom) lines and control (null) from T3 generation. Triacylglycerol (a), polar lipids (b), free fatty acids (c), other acyl lipids (d). The results are shown as the mean ± standard deviation from three biological replicates. Ins, gene inserts. Asterisks indicate significant differences as compared to control according to Fisher’s test with a significance threshold of *P* ≤ 0.05.

**Additional file 7: Figure S5.** Protein content on dry weight (dw) basis in endosperm (including starchy endosperm, aleurone cells, seed coat and pericarp) of developing grains from selected homozygous (hom) *AsWRI1*-wheat lines (with one or 12 gene inserts) and control (null) from T3 generation. The results are shown as the mean ± standard deviation from three biological replicates. No significant differences as compared to control according to Fisher’s test with a significance threshold of *P* ≤ 0.05 was observed.

**Additional file 8: Figure S6.** Net accumulation of ^14^C in wheat grain endosperms (including starchy endosperm, aleurone cells, seed coat and pericarp) at different time points (T) 0–192 h after given ^14^C -sucrose pulse to detached spikes of wheat. The results are shown as the mean ± standard deviation from three biological replicates. Asterisk indicates significant difference as compared to control according to Fisher’s test with a significance threshold of *P* ≤ 0.05.

**Additional file 9: Figure S7.** Net accumulation of ^14^C in different lipid classes in grain endosperms (including starchy endosperm, aleurone cells, seed coat and pericarp) at different time points (T) 0–192 h after given ^14^C-sucrose pulse to detached spikes of *AsWRI1*-wheat (with 12 gene inserts) and corresponding null. Triacylglycerol (a); polar lipids (b), free fatty acids (c), diacylglycerol (d), other remaining acyl lipids (e). The results are shown as the mean ± standard deviation from three biological replicates. Asterisks indicate significant differences as compared to control according to Fisher’s test with a significance threshold of *P* ≤ 0.05.

## Data Availability

The plant material and the datasets supporting the conclusions of this article are available from the corresponding author on reasonable request.

## Abbreviations

dpa: Days post anthesis
dw: Dry weight
FAME: Fatty acid methyl ester
TAG: Triacylglcyerol
TPM: Transcripts Per kilobase Million
WRI1: WRINKLED1
