# Supplementary figures and images for: Transitions in wheat endosperm metabolism upon transcriptional induction of oil accumulation by oat endosperm WRINKLED1
Source: BMC Plant Biol. 2020 May 25;20:235. doi: 10.1186/s12870-020-02438-9 (PMC7249431; doi:10.1186/s12870-020-02438-9)

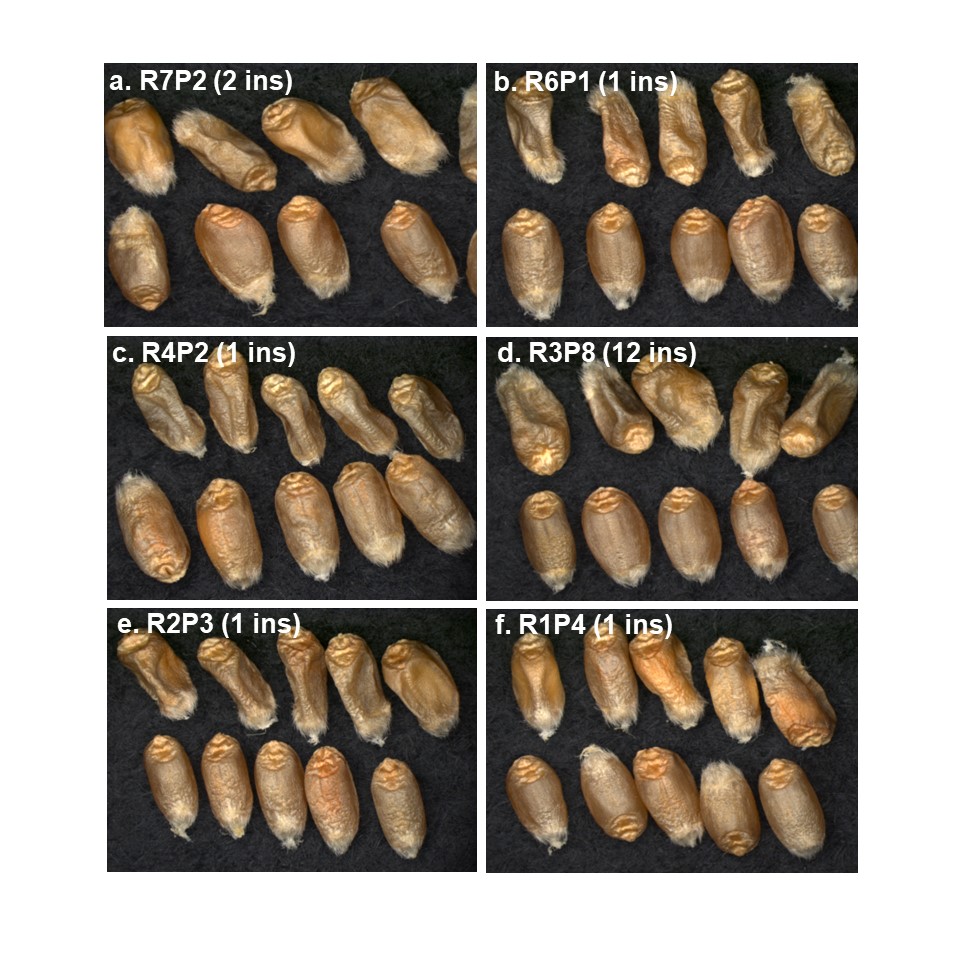

Supplement: Supplementary file 3 — Additional file 3: Figure S1. Photos show the wrinkled seed phenotype of homozygous AsWRI1-wheat lines (upper row in each image) of T2 generation as compared to in their corresponding nulls (bottom row in each image) coming from six individual transformation events (a-f). Number of gene inserts (ins) are given in parentheses. [file 12870_2020_2438_MOESM3_ESM.jpg]

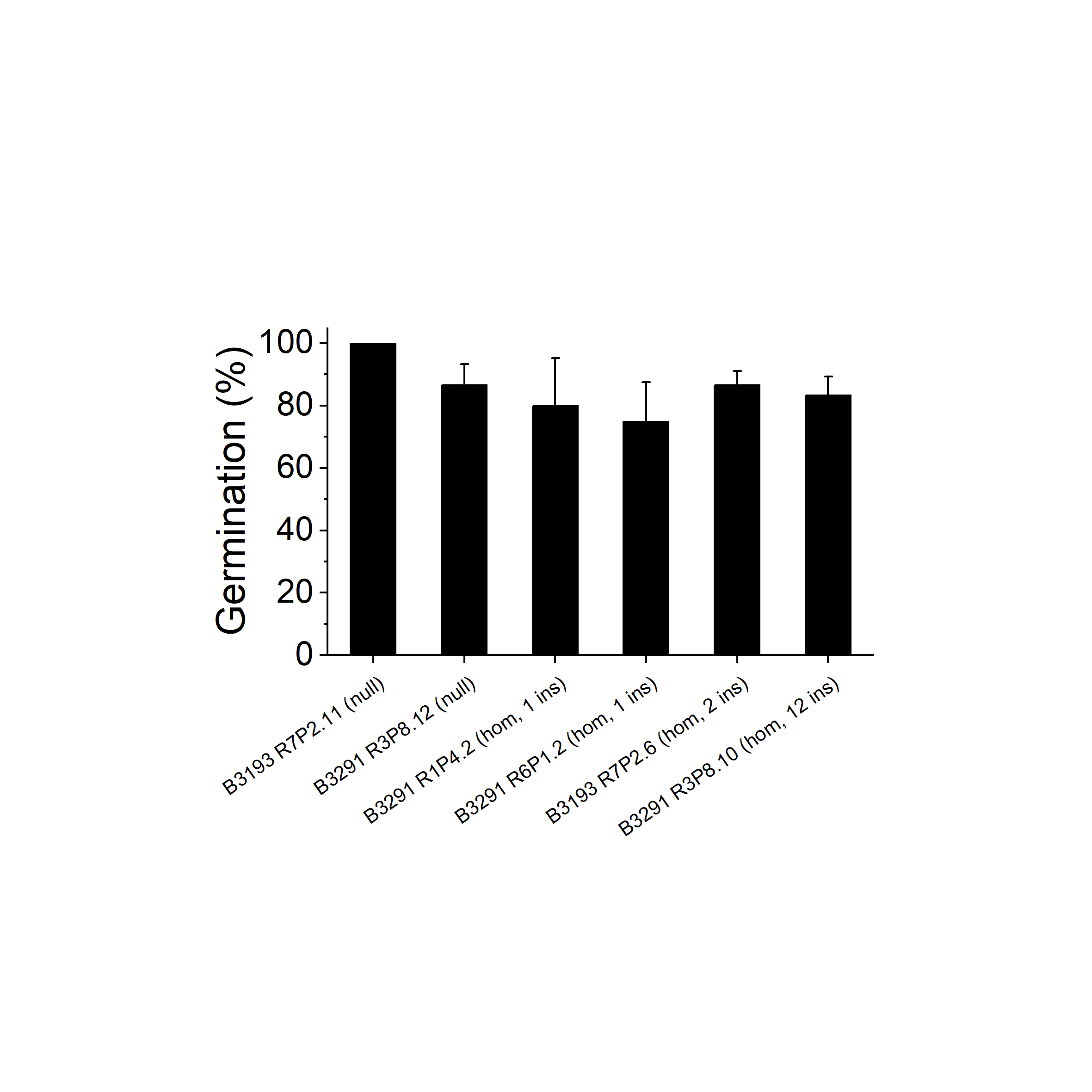

Supplement: Supplementary file 4 — Additional file 4: Figure S2. Germination tests of AsWRI1-wheat grains in % of total grains. The results are shown as the mean ± standard error from three biological replicates, each consisting of 20 seeds. The generalized linear model showed that there were no real differences in the proportion of germination across the types (p = 0.110, F-test) or between the lines having accounted for type (p = 0.427, F-test). Number of gene inserts (ins) are given in parentheses. Hom, homozygous. [file 12870_2020_2438_MOESM4_ESM.jpg]

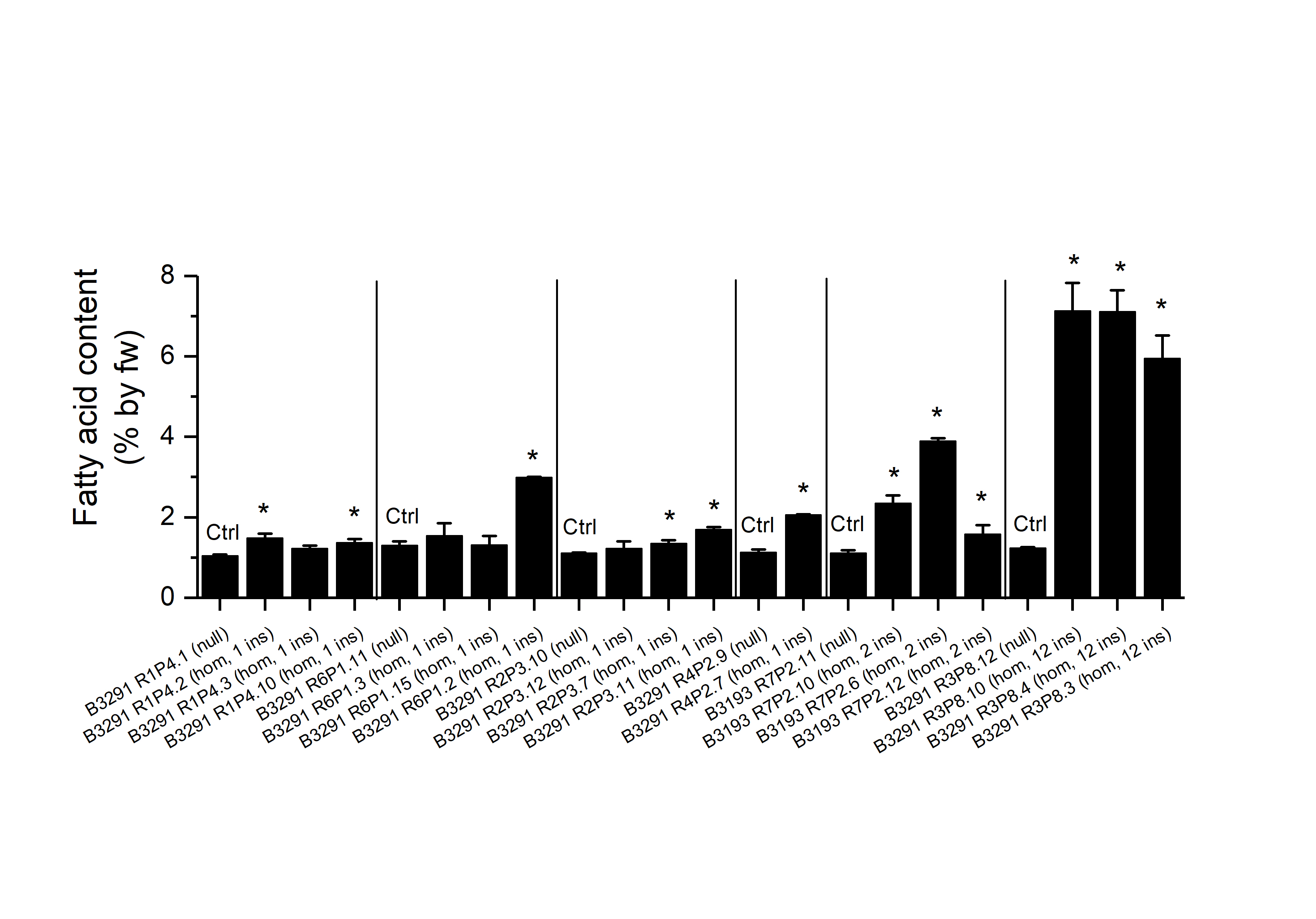

Supplement: Supplementary file 5 — Additional file 5: Figure S3. Fatty acid content of total lipids on fresh weight (fw) basis in the endosperm (including starchy endosperm, aleurone cells, seed coat and pericarp) of mature AsWRI1-wheat grains from 16 homozygous (hom) lines (coming from six individual transformation events) and their corresponding nulls (controls; Ctrl) from T2 generation. The results are shown as the mean ± standard deviation from duplicate samples (á 3 grains) from seed batches from each line. Ins, gene inserts. Asterisks indicate significant differences as compared to control according to Fisher’s test with a significance threshold of P ≤ 0.05. [file 12870_2020_2438_MOESM5_ESM.jpg]

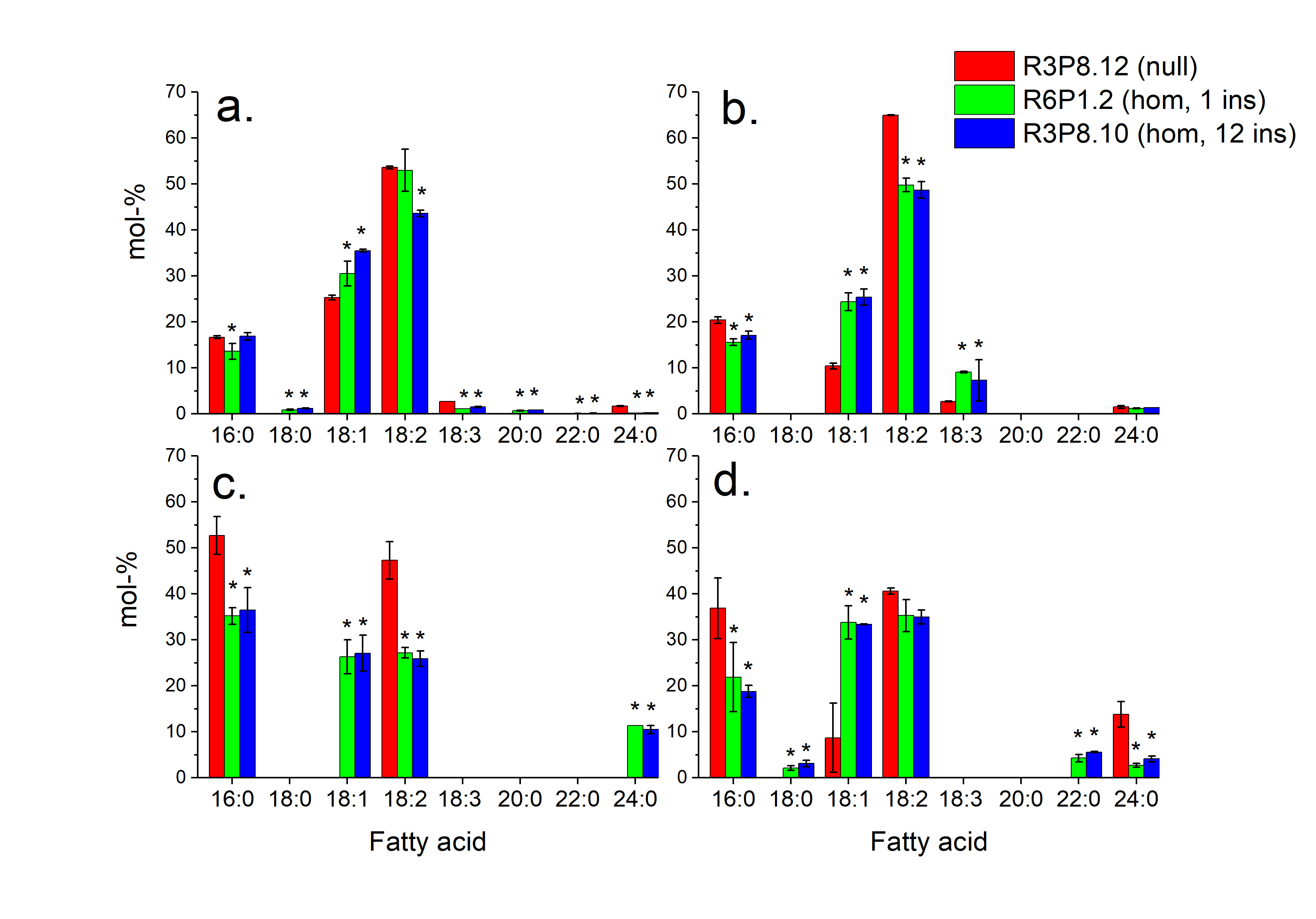

Supplement: Supplementary file 6 — Additional file 6: Figure S4. Fatty acid profiles of different lipid classes in endosperm (including starchy endosperm, aleurone cells, seed coat and pericarp) of mature AsWRI1-wheat grains from selected homozygous (hom) lines and control (null) from T3 generation. Triacylglycerol (a), polar lipids (b), free fatty acids (c), other acyl lipids (d). The results are shown as the mean ± standard deviation from three biological replicates. Ins, gene inserts. Asterisks indicate significant differences as compared to control according to Fisher’s test with a significance threshold of P ≤ 0.05. [file 12870_2020_2438_MOESM6_ESM.jpg]

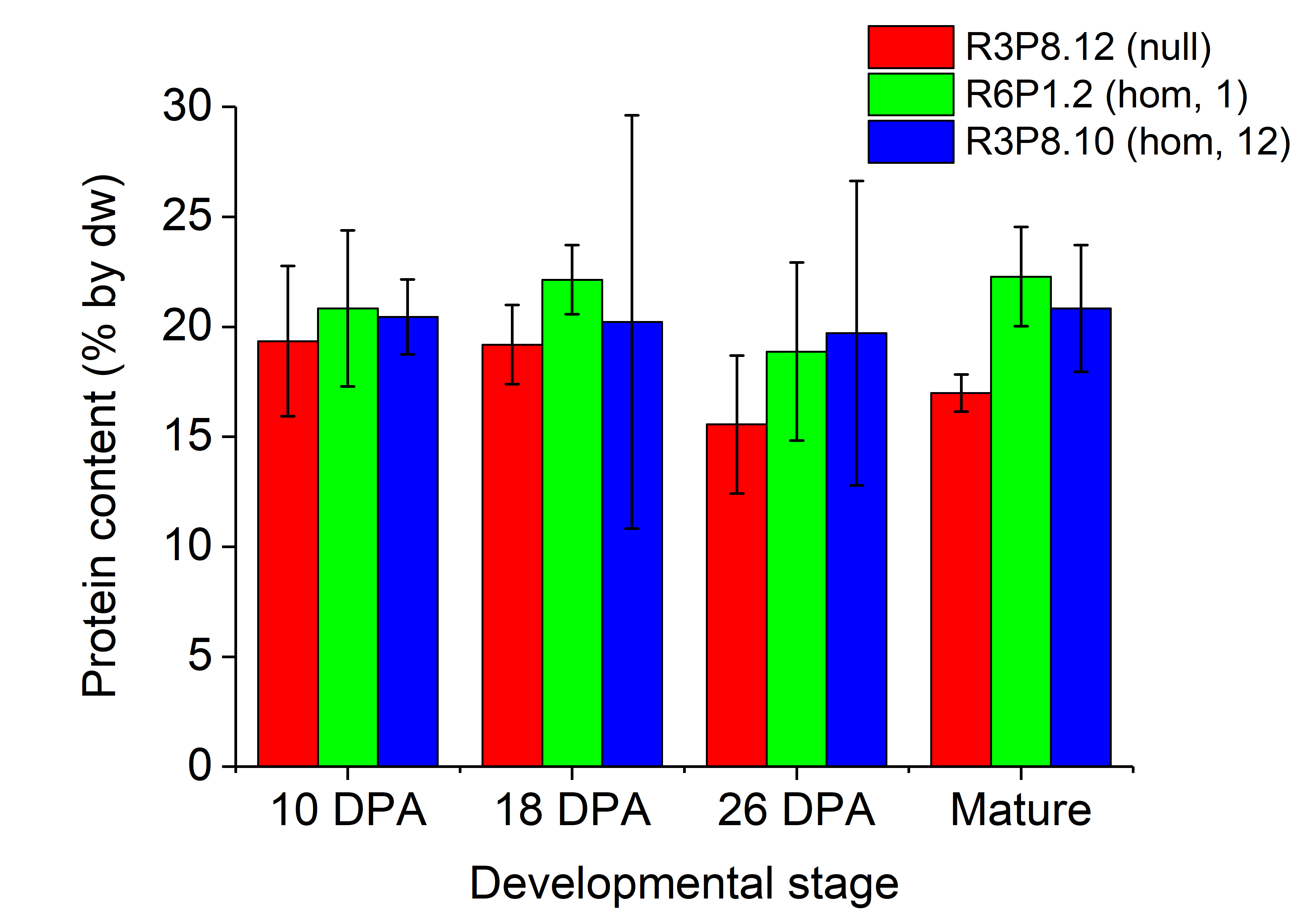

Supplement: Supplementary file 7 — Additional file 7: Figure S5. Protein content on dry weight (dw) basis in endosperm (including starchy endosperm, aleurone cells, seed coat and pericarp) of developing grains from selected homozygous (hom) AsWRI1-wheat lines (with one or 12 gene inserts) and control (null) from T3 generation. The results are shown as the mean ± standard deviation from three biological replicates. No significant differences as compared to control according to Fisher’s test with a significance threshold of P ≤ 0.05 was observed. [file 12870_2020_2438_MOESM7_ESM.jpg]

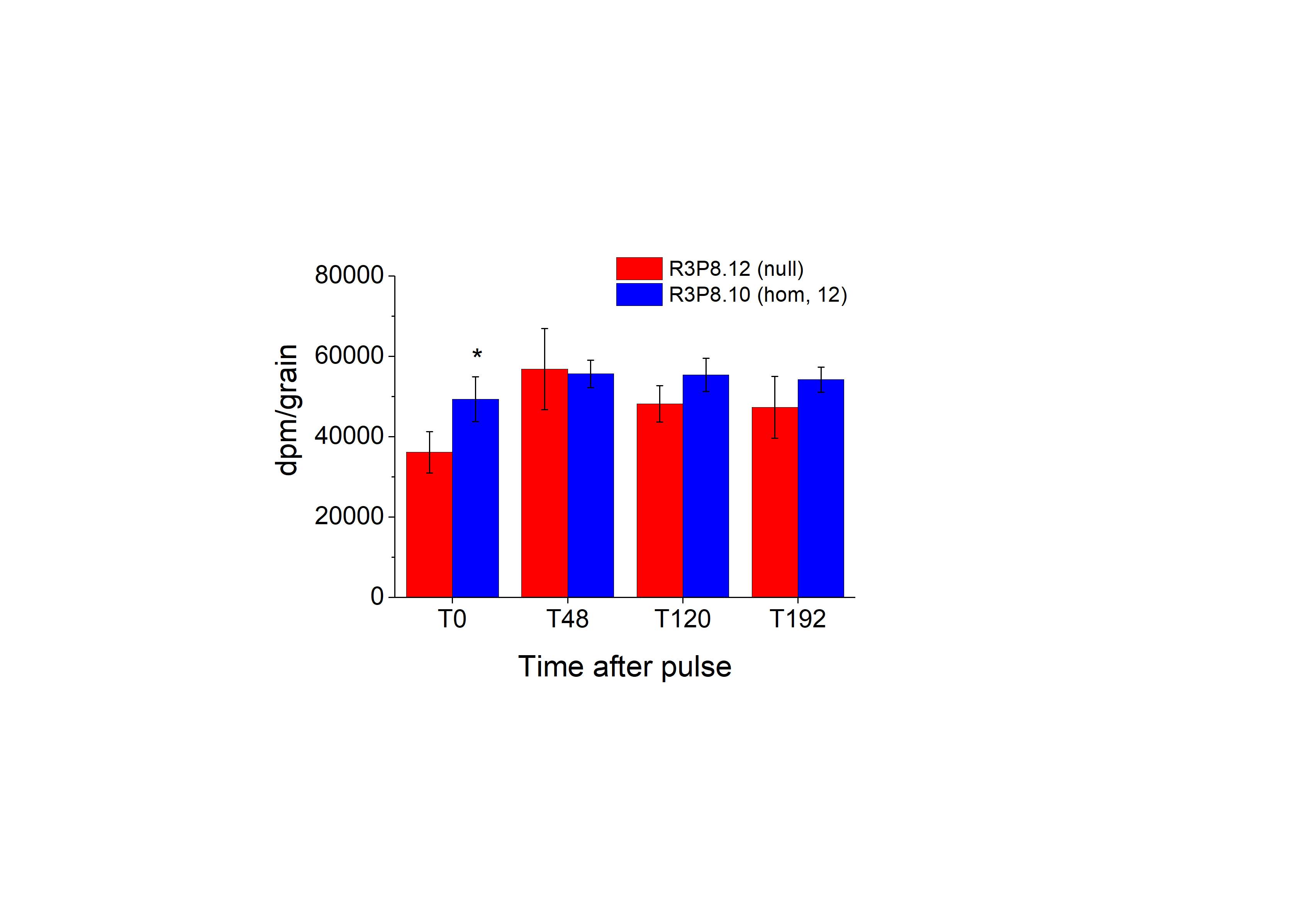

Supplement: Supplementary file 8 — Additional file 8: Figure S6. Net accumulation of 14C in wheat grain endosperms (including starchy endosperm, aleurone cells, seed coat and pericarp) at different time points (T) 0–192 h after given 14C -sucrose pulse to detached spikes of wheat. The results are shown as the mean ± standard deviation from three biological replicates. Asterisk indicates significant difference as compared to control according to Fisher’s test with a significance threshold of P ≤ 0.05. [file 12870_2020_2438_MOESM8_ESM.jpg]

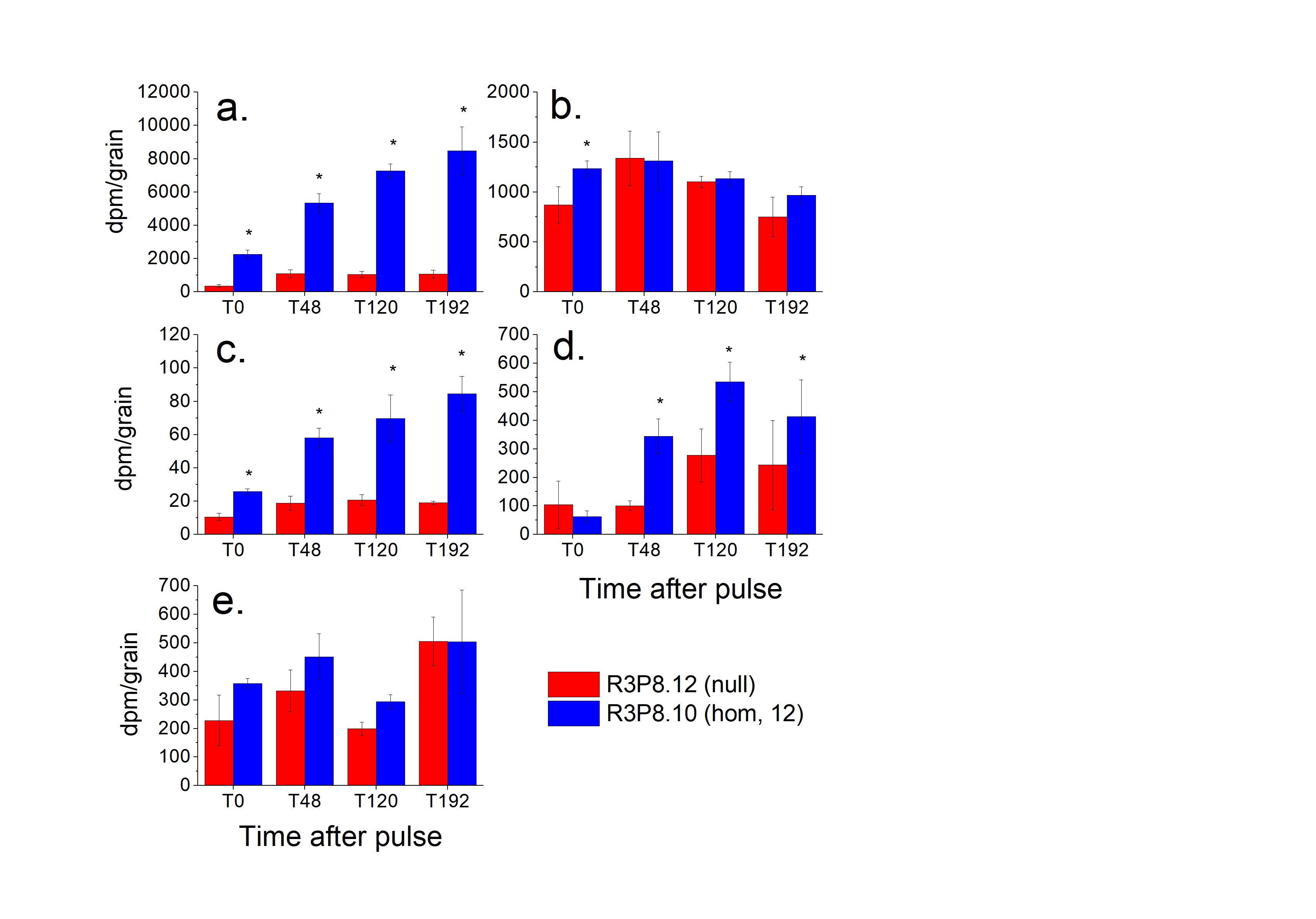

Supplement: Supplementary file 9 — Additional file 9: Figure S7. Net accumulation of 14C in different lipid classes in grain endosperms (including starchy endosperm, aleurone cells, seed coat and pericarp) at different time points (T) 0–192 h after given 14C-sucrose pulse to detached spikes of AsWRI1-wheat (with 12 gene inserts) and corresponding null. Triacylglycerol (a); polar lipids (b), free fatty acids (c), diacylglycerol (d), other remaining acyl lipids (e). The results are shown as the mean ± standard deviation from three biological replicates. Asterisks indicate significant differences as compared to control according to Fisher’s test with a significance threshold of P ≤ 0.05. [file 12870_2020_2438_MOESM9_ESM.jpg]
